# Confronting death at birth: dialogues and solutions from health teachers and students

**DOI:** 10.1590/1980-220X-REEUSP-2025-0381en

**Published:** 2026-04-10

**Authors:** Isadora Ferrante Boscoli de Oliveira Alves, Margarete Maria de Lima, Juanah Oliveira Debetio, Kênia Lara Silva, Maria Ligia dos Reis Bellaguarda, Roberta Costa

**Affiliations:** 1 Universidade Federal de Santa Catarina, Programa de Pós-graduação em Enfermagem, Florianópolis, SC, Brazil.; 2 Universidade Federal de Minas Gerais, Escola de Enfermagem, Belo Horizonte, MG, Brazil.

**Keywords:** Interprofessional Education, Faculty, Students, Attitude to Death, Obstetrics

## Abstract

**Objective:**

To identify solutions to prepare health professionals to deal with death in the context of birth based on the experiences of teachers and students.

**Methods:**

A qualitative, problematizing study was conducted with 10 teachers and 2 students from the medicine, nursing, and psychology courses at a university in southern Brazil. Data collection was carried out through focus groups, organized according to the stages of Charles Maguerez’s problematization arc. The analysis followed Minayo’s operationalization and Paulo Freire’s theoretical framework.

**Results:**

Health professionals reported difficulties in welcoming different types of grief, associated with gaps in education, institutional limitations, and a lack of spaces for listening. As solutions, they highlighted the cross-cutting inclusion of the theme in undergraduate courses, the creation of spaces for emotional support, the encouragement of interdisciplinary groups, and the strengthening of professional leadership in the face of ethical and care challenges.

**Conclusion:**

The sharing of experiences revealed educational and transformative potential, highlighting the urgency of curricular and institutional changes to improve the quality of care.

## INTRODUCTION

Death marks the end of life and is characterized as a biological and social event, capable of evoking different meanings and emotional reactions in those who face it. Although it is part of the cycle of life, death is often feared by most people, especially when it occurs violently or abruptly. In this sense, unexpected deaths, such as in the context of birth, often provoke reactions of shock, anger, and deep suffering in those who experience them^(1,2,3)^.

According to data from the Pan American Health Organization (PAHO), every day approximately 830 women die from preventable causes related to pregnancy and childbirth worldwide, with 99% of these deaths occurring in developing countries. In Brazil, these rates increased by 94.4% during the COVID-19 pandemic, reaching 107.53 cases per 100,000 live births (LB) in 2021^(4)^. As for neonatal deaths, which correspond to deaths within the first 28 days of a baby’s life, the rate is estimated at approximately 17 deaths per 1,000 LB in 2019, equivalent to 6,700 deaths per day. In Brazil, the average recorded was 12.4 deaths per 1,000 LB in 2018, an improvement compared to the 1940s, when rates reached 146.6 deaths per 1,000 LB^(5,6)^. It is therefore clear that deaths related to childbirth are common worldwide and require attention and preparation to address them.

Authors point out that society often attributes to doctors the responsibility of preventing death from occurring. Thus, it is common for health education to focus on developing technical skills for diagnosis and clinical interventions in future professionals, sometimes neglecting the psychosocial aspects involved in practice and in dealing with patients. Consequently, when faced with subjective experiences, such as those involved in coping with death, these professionals find themselves without the necessary tools and skills to deal with the situation^(7,8,9)^.

Studies indicate that unprepared or inexperienced professionals tend to adopt negative attitudes when faced with a situation of death, in order to avoid their own emotions. Thus, these professionals assume a mechanized and apparently cold attitude when dealing with patients and their families, creating a distant and unconnected relationship that contradicts the principles of the Unified Health System (SUS) (universality, comprehensiveness, and equity) and the National Humanization Policy. On the other hand, properly trained professionals demonstrate greater ability to manage their emotions and offer support to families, helping them to overcome their loss in a healthier way^(2,8,9,10,11)^.

Elisabeth Kübler-Ross (1926–2004), a pioneer in palliative care, affirmed the importance of health professionals knowing their stance on death before providing care to family members, highlighting the impact that their actions have on patients’ ability to overcome grief. The psychiatrist emphasized the need for education courses to develop students’ awareness of the reality of death itself, in order to make them reflect on their own finitude and demystify the topic in their professional practice^(1)^.

In view of this, research shows that undergraduate courses in the health field integrate content related to death into their curricula. However, they tend to take a technical approach, focused on aseptic procedures, filling out death documentation, and the conduct that professionals should have at the time of death.

Thus, they do not prepare students to handle these situations in a welcoming, empathetic, and humane manner, negatively impacting the experience of family members and the exercise of professional practice^(8,9,12,13)^.

Therefore, based on the assumption that hospitals have become the primary places for childbirth; that health professionals regularly encounter the process of death in this context and do not have the technical and emotional preparation to deal with this situation; and that their actions have a direct impact on the lives of families, the objective is to identify solutions to prepare health professionals to deal with death in the context of birth based on the experiences of teachers and students.

## METHOD

### Study Design

This is a qualitative study with a problematizing approach, based on the theoretical framework of Paulo Freire^(14)^. The research is part of a mixed-transformative macro-project entitled “Pedagogical proposal for dealing with death and grief in the context of birth in the education of health professionals,” which aimed to collectively construct an educational proposal focused on preparing health professionals to deal with death and grief in the context of birth. The final report of the study was prepared in accordance with the Consolidated Criteria for Reporting Qualitative Studies (COREQ): 32 checklist.

### Location

In order to develop solutions to prepare health professionals to deal with death in the context of birth, based on the experiences of teachers and students, the scenarios chosen for this research were the undergraduate health courses at a public university located in southern Brazil. The choice took into account the institution’s more than 60 years of history and its ranking among the five best universities in the country in the 2019 General Course Index (IGC), which placed it among the educational institutions with a standard of excellence in the Ministry of Education classification.

Although the institution offers several undergraduate courses related to the health field, for this study, only Nursing, Medicine, and Psychology were considered, due to their significant role in the context of birth and because they deal directly with the process of coping with death and mourning of families in this scenario. These three areas also stand out as the main ones responsible for scientific production on the topic, as pointed out by the findings of the literature review.

### Population, Selection Criteria, and Sample Definition

The population investigated consisted of a total of 264 students enrolled in the last two phases of the undergraduate courses in Nursing, Medicine, and Psychology at the aforementioned university (58 in Nursing, 134 in Medicine, and 72 in Psychology). However, only 38 students were included in this study, as they had participated in a previous stage of the macroproject, which consisted of completing a questionnaire related to the approach and preparation received in education to deal with situations of death and grief. Forty-one teachers mentioned by these students as potential participants in this questionnaire were also considered, as they taught subjects or conducted research on maternal and child health and/or the processes of death and mourning. Invitations were sent via institutional email and included: a letter of introduction to the study, the Free and Informed Consent Form (FICF), and a characterization form.

The eligibility criteria were: being a faculty member and teaching undergraduate courses in Nursing, Medicine, or Psychology at the selected university, with at least one year of experience; or being an undergraduate student enrolled in the last two phases of the aforementioned courses and having participated in the first phase of the macroproject. Temporary faculty members were excluded.

### Data Collection

Data collection was carried out through focus groups, led by a facilitator—the principal investigator, at the time a doctoral student in nursing with experience in qualitative research, responsible for presenting the topic and guiding the discussions in an active and participatory manner— and a rapporteur—also a doctoral student in nursing, with experience in interviews and group activities, who observed and recorded data related to the participants’ interaction in a field diary, in a non-participatory manner—in addition to the participants themselves^(14)^. Because they belonged to the institution being researched, both the facilitator and the rapporteur already knew most of the participants, albeit superficially.

To adapt the research to the availability of the guests, three collection moments were organized: a virtual meeting, a face-to-face meeting, and a validation stage. Both the dynamics of the virtual meeting and the face-to-face meeting were repeated three times, on different dates, totaling six focus groups (three *online* and three face-to-face). To this end, a script adapted from Charles Maguerez’s problem-solving arc was used to ensure the methodological rigor of the research. The script followed these steps: 1) Recognition of reality; 2) Listing of key points; 3) Theorization; 4) Solution hypotheses; and 5) Application to reality. It should be noted that each participant attended only once at each stage of data collection^(15)^.

The virtual meeting was held using the *Google Meet* ^®^ platform. On the scheduled day, participants were welcomed by the facilitator and the rapporteur, who gave a brief presentation of their credentials, the research objectives, and the guidelines for conducting the group on that day. The participants were then informed about the recording of the meeting, which began only after everyone had given their consent.

To begin the development phase, an “icebreaker” activity was conducted, based on three trigger questions: “a) What does death represent and how do I deal with it in my professional practice?; b) How do I perceive the experience of death in the context of birth?; and c) How do I assess the preparedness of health professionals to deal with death in the context of birth?”. Participants were then asked to enter images and/or words representative of their answers into the Jamboard^®^ application within 15 minutes. At the end, each member presented their reflections, beginning the first stage of Charles Maguerez’s arc (Recognition of reality).

From there, participants identified the points of conflict involved in the issue (2nd stage of the arc); theorized about their occurrence (3rd stage); and articulated ideas compatible with their reality of action to solve them (4th and 5th stages). The elements brought by the participants in each stage were recorded in the form of topics in the application itself, allowing simultaneous monitoring and awareness by all. At the end, the facilitator thanked the participants for their participation and announced the dates of the second meeting. The activities on *Jamboard* ^®^ were photographed and used, together with the recordings, to prepare for the face-to-face meeting.

The second moment of group interaction took place in person at the university itself, following all the safety measures recommended by the Ministry of Health to contain the spread of Covid-19, which were still in force at the time. The facilitator again welcomed the participants and informed them about the recording. This was followed by a new “icebreaker” activity, in which images of the activities carried out in the virtual groups on the Jamboard^®^ app were presented, followed by a summary of the main topics discussed. The data was displayed on a board so that participants could review, correct, supplement, or delete information until consensus was reached among all and the data was saturated.

As the face-to-face meetings also took place separately, the principal investigator promoted an additional stage of validation of the content produced. To this end, a file containing the systematized data from the two collection moments was sent to the participants by email so that they could validate and/or propose changes to the content within 15 working days. In the end, only textual corrections were requested, thus ending the validation phase.

The average duration of each group, both online and in person, was approximately two hours. All recordings were transcribed by the principal investigator. Data collection took place between August 2022 and March 2023.

### Data Analysis and Processing

Data processing followed the operational proposal of Minayo^(14)^, in which the fundamental determinants of the field and its actors were mapped; the empirical factors observed were interpreted, grouping them into categories; and an inflection analysis was performed to grasp the concrete and abstract content of the discourses in order to understand the meanings that permeate them. Next, the data, previously organized based on the stages of the problematization arc, were analyzed in light of Paulo Freire’s theoretical framework, resulting in categories and subcategories of meaning.

### Ethical Aspects

To preserve the anonymity of the participants, the names of the teachers were replaced by the acronym “DO” and those of the students by “DI,” followed by a number corresponding to the order of participation (DI1, DO2, DI3). The study followed the guidelines of Resolutions No. 466/2012 and No. 510/2016 of the National Health Council, as well as the guidelines of the National Research Ethics Commission (CONEP) and Official Letter No. 2/2021, which provides for research procedures in virtual environments. The research was submitted to the university’s Research Ethics Committee and approved under opinion No. 5,287,016, having been initiated only after the signing of the Free and Informed Consent Form by the participants.

## RESULTS

Forty-one faculty members were invited to participate in the study, of whom 10 actually attended the data collection stages. Of these, eight belonged to the Nursing course, one to Medicine, and one to Psychology. All participants were women with doctoral degrees. Regarding marital status, three were single, two were divorced, and the rest were married. The average length of service at the university among the participating teachers was approximately 8.2 years.

Regarding the students, of the 38 contacted, 11 expressed interest in continuing to participate in the research. However, only two students were able to attend the meetings: one from the Nursing course and the other from Medicine. Both were female, one married and the other single, aged between 22 and 26. In total, 12 people participated in the data collection stage: 10 teachers and 2 students.

Considering the phases of the problematization arc and Paulo Freire’s framework, the study data were organized into two main categories of meaning, subdivided into four and five subcategories, respectively: 1) Recognizing reality and its challenges: a) The meaning of death in the context of birth differs from other scenarios; b) Welcoming different types of grief; c) Specialized knowledge and emotional availability; d) Institutional limitations. 2) Identifying viable solutions: a) Making death a cross-cutting theme; b) Gradual and safe contact with the experience; c) Spaces for the expression of feelings; d) Multidisciplinary study groups; e) Encouraging active involvement: the professional as an agent of change.

### Recognizing Reality and its Challenges: The Meaning of Death in the Context of Birth Differs from Other Scenarios

The data indicate that the experience of death in the context of birth impacts health professionals in a unique way, differing from other losses. This is due to the symbolic meaning of the beginning of life, combined with the frequent association with social inequalities and failures in care, which gives neonatal/maternal death a specific emotional charge.

*Death in the context of birth is almost destabilizing [...] when it happens in an obstetric center [...] and that whole space is prepared for life, and suddenly there is a situation of death, it is something very delicate and sometimes difficult to deal with [...] so much so that the vast majority flee [...] no one wants to have that responsibility.* (DO2)

*We realize that death in the context of birth is directly related to social indicators. It reflects the quality of healthcare for this population, the intersectionalities, because we know that more black, indigenous, and lower-class women die [...] and even though we have public policies [...] we know that we are very far from achieving the goals, especially in relation to maternal mortality [...]* [which] *are, for the most part, preventable deaths.* (DO6)

### Recognizing Reality and its Challenges: Welcoming Different Types Of Grief

The context of birth involves different types of grief, which vary according to the moment of death (early pregnancy, late pregnancy, postpartum), the type of death (maternal, fetal, or neonatal), and its causes (spontaneous, predicted or expected, errors in early identification of complications and management, among others). In addition, grief is experienced by different individuals (fathers, mothers, siblings, grandparents, health professionals, among others) and is associated with the significance that the deceased had for each family member.

*After the curettage, we end up not dealing [with the woman] [..] She will often be hospitalized for a while before the procedure [...] experiencing this grief, experiencing this loss, and no one sees it. It’s real, there’s no one there to give her support because: “well, it’s very early on, her belly hasn’t even grown yet.* (DO2)

*Once I accompanied a maternal death [...] and then I saw the little sister arrive at ten years old and ask to hold* [the baby]*, and she was already assuming the role of mother, a ten-year-old child. She also lost her mother, you know?* (DO9)

*[...] the death of a baby is when you lose a dream. [...] Now, when we lose our mother, I think it’s very complicated, especially for the father who is left behind and if there is still a baby. [...] It’s as if a story has been erased. So it hurts in every way, the death of an elderly person, a baby, a child, a teenager will hurt, but I think these are pains that have different connotations because they will have different dreams, different involvements, different expectations.* (DO11)

### Recognizing Reality and its Challenges: Specialized Knowledge and Emotional Availability

The emotional availability required to deal with the death and suffering of families represents a significant challenge for professionals, as it demands careful management of personal feelings, experiences, and beliefs, as well as mastery of specific technical skills.

*Dealing with someone who deals with death is knowing how to silence your own experience of death [...] I would like someone to tell me that I am still young* [to have a child]*, but empathy is not about that, empathy is not about doing what you would want done for you, empathy is doing what that person needs done for them. So understanding this dynamic of the person’s context, silencing it and doing it, is fundamental.* (DI3)

*I see that there are basic recipes or protocol behaviors that we follow [...] For example, if you have a mother who has lost a baby, you don’t put her in the same room with the other mothers [...]. When a baby is born* [dead]*, you always encourage the mother to hold that baby and say goodbye [...] But sometimes [...] her answer is no. Then you wait, give her some time [...] Then, often, they change their minds [...]. So, we have ways of trying to ease this pain [...] but I think professionals need to look beyond, to try to understand what people are experiencing.* (DO9)

*We had a very difficult experience with a mother who knew that her baby would not survive many hours after birth, and she had other children. So, the other children, together with their father and mother, wrote a little letter [...] then, when she had the baby, the baby came to us, alive, for us to let him go. [...] We put on all the little clothes [...] and read the letter to him* [baby] *[...]* [and] *held him in our arms [...]. So it’s not easy,* [because] *you’re saying goodbye to a little child who isn’t even yours!* (DO11)

### Recognizing Reality and its Challenges: Institutional Limitations

For the research participants, the high turnover of professionals in health institutions, motivated by low salaries, leads to work overload, mechanization of care, and weakening of teamwork. Consequently, these factors hinder the humanized approach to grief, in addition to increasing the frequency of deaths in the workplace.

*Last week we were talking about how maternal mortality is increasing in the statistics and [...]* [this] *couldn’t happen, because maternal mortality is a mistake, it’s a failure [...] And what do we see? There are a lot of staff changes [...] because the salary isn’t worth it [...]* [the amount is] *a third of what you earn working for the state, so no one stays.* (DO2)

*I work a lot in primary care and* [there] *this is something even more* [distant]*, because it seems that we already have to move on to another subject: “let’s refer them to a psychologist, this is no longer my responsibility,” you know? It’s an escape [...] So, in the hospital, we already have little preparation [...], [but] I think that in primary care,* [it’s] *even less.* (DO6)

*We hear a lot: “I don’t even have time to take care of the living, am I going to take care of those who have died?” Not that those who have died are unimportant, but I don’t have time. And then I think it forms a vicious cycle [...] then when I have time, I don’t even go because I don’t even do that in the course of the things I need to do.* (DO12)

### Identifying Viable Solutions: Making Death a Cross-Cutting Theme

Considering the scope of the theme and its impact on healthcare, the participants concluded that the theme of death should be addressed in a cross-cutting manner in health education.

*I think that undergraduate courses should always have, in a cross-cutting manner,* [and in] *all disciplines, some content related to death and caring for people and families, because it is something that has to be constant. [...] Having a cross-cutting proposal that addresses the various types of death is not a specialization course, but it is the minimum that students need to have in terms of concepts, experiences, and pedagogical strategies in order to mature.* (DO7)

*In the context of birth, when abortion occurs [...] there is also illegal abortion. [...]* [And] *these people come to us in the services and have also gone through a process of mourning, death, loss, social judgment [...] of psychosocial health. So, if I think about working on this in education, I think it is a bioethical* [cross-cutting] *theme that is part of it.* (DO8)

### Identifying Viable Solutions: Gradual and Safe Contact with the Experience

In terms of learning, the importance of providing gradual and realistic contact with the theme of death was emphasized, so that students understand its complexity and develop the necessary skills to face it.

*I saw children being born without brains [...], I saw mothers who knew that their child was already dead and were waiting for the time of delivery to give birth [...] and I experienced this during my undergraduate studies. How did I benefit from this? There were two obstetric nurses who accompanied me during my internship [...]* [and] *they gave me all the support I needed, not only emotionally, but also technically. I accompanied them and did everything together with them. So this was very important and also brought me professional maturity.* (DO7)

*Everyone remembers the first death they witnessed [...] and that is what will give us confidence, knowledge, and reflection. [...] Those who have lost someone remember that the words really stay in your memory [...], what was good [...] and the words that made [us] break down. So, you have to be very careful when you speak.* (DO9)

### Identifying Viable Solutions: Spaces for the Flow of Feelings

The importance of support groups was highlighted, in which teachers and students can express their feelings related to grief, share experiences, and participate in joint continuing education activities, aiming at the development of technical and sensitive skills for the proper handling of situations involving death and grief.

*The institution, considering its resources, finds it very difficult to have a professional who is linked to you. A psychologist will not be able to support you because it is against their own principles to have professional ties there.* [Search] *an outside professional, most people don’t have the money to pay for it. And neither does the institution. So, what have I always seen? You create your small discussion groups about the problems you have experienced [...] to provide both theoretical support and an exchange of experiences for new challenges. But the vast majority of groups are always like this: “I don’t have time, I don’t have time.* (DO7)

*I was very surprised to discover through my thesis [...] that professionals in general have a lot of difficulty dealing with death, especially when it comes to breaking the news [...] to family members[...] so I realized [...] that professionals suffer a lot [...]. And when these deaths are a constant in your work, the suffering is even greater. [...] So, I think there needs to be support for professionals who also don’t have moments of reflection*. (DO10)

### Identifying Viable Solutions: Multidisciplinary Study Groups

It was also suggested that multidisciplinary exchanges be held at universities, with the aim of promoting understanding of different perspectives on death and how to deal with it, helping to overcome any difficulties inherent in each area of activity.

*I think the support of colleagues themselves, at least in our department [...] you have a group of teachers who have regular meetings [...] everyone has a doctorate, a post-doctorate, many courses... I see a very healthy exchange of experiences, plans, and how to approach students.*

*It may not give you 100% support, but I understand, from my point of view, that it is healthy and helps a lot. (DO7) This conversation circle here today, what a good thing even for us who are participating [...]. This should happen more often, we need to talk more about this subject in society in general, also in education and care.* (DO10)

### Identifying Viable Solutions: Encouraging Active Involvement: The Professional as an Agent of Change

Two participants emphasized the importance of promoting the “agency” of health professionals in the education process, encouraging them to take a proactive stance in the face of conflicts between the education received, the needs of the population served, and current institutional regulations.

*Something I would add would be to understand institutional limitations [...] “You can’t come in,” “you can’t see,” “you can’t take” [...] it’s okay that the whole family can’t see a child who is in the ICU [...] but is it okay that we have to send a child to another place [...] and take them out of that small crib? [...] I think understanding institutional limitations in these terms [...] is important [...]* [to] *realize your potential to transform these rules, you know?* (DI3)

*We are trained to work in a legal manner and then the institution’s rules come along [...]. You freeze, because you’re a good guy. You learned to follow rules, not only because they are necessary, but because they also speak to our social contract to maintain a cohesive practice, right? [...] Agency means precisely that you create rules from a perspective [...] of dialoguing with these rules and creating a new way of life, a new way of caring.* (DO5)

## DISCUSSION

It is observed that teachers and students experience situations of death in the context of birth throughout their education. However, because of the association of this scenario with the beginning of life, the meaning of death acquires specific connotations that influence how both professionals and the bereaved cope. To this end, welcoming of grief in this context presents complexities, as it involves symbolic factors, beliefs, and social inequalities that are often neglected by education processes, resulting in insufficient preparation and limited emotional availability to adequately conduct the loss process according to the unique needs of each bereaved person (statements from the first and second subcategories).

Studies point out that perinatal loss is often not recognized by society, since parents do not have the opportunity to live with their child for a long time. This fact fosters the belief in the collective imagination that suffering is less in these cases and that parents do not need specific care^(8,9,11)^. Consequently, parents in the process of loss are prevented from showing their grief in public, while at the same time having their motherhood/fatherhood invalidated by the environment, including by health professionals.

Effective welcoming requires practices based on dialogue and the building of bonds. A booklet on death education suggests that health professionals need to consider six elements to understand a person’s grieving process: 1) who the deceased was in the life of the bereaved and what they represented; 2) the nature of the emotional bond between them; 3) the circumstances of the death and how the bereaved person received the news; 4) history of previous complicated grief; 5) the personality of the bereaved person; 6) and the social factors involved, such as cultural, ethnic, and religious influences and the presence of a support network^(16)^. Therefore, it makes explicit the importance of professionals developing skills of “silence and empathy” to listen to the pain of others, in order to become capable of “looking beyond the circumstances” and providing assistance beyond pre-established institutional protocols (statements from the third subcategory).

The implementation of complex care practices, such as Welcoming Grief Counseling, requires time, education, and coordination among health teams. Consequently, such practices are directly affected when there is work overload, professional turnover, and mechanization of care (factors mentioned in the fourth subcategory). A descriptive study conducted in 35 family health units in a medium-sized state in Brazil identified that high staff turnover and lack of specialized education impact the services provided by teams, resulting in unsatisfactory evaluations by users in almost all items included in the Primary Care Assessment Tool (PCATool-Brazil)^(17)^.

Another study^(18)^, conducted with 166 nurses, indicated that turnover in health institutions is related to five factors: management support, reward, physical comfort, control/pressure, and team cohesion. The data indicate that increasing “rewards” is the most effective factor in reducing turnover intentions, while decreasing physical comfort and low team cohesion increase them, which is consistent with the statements in the “institutional limitations” subcategory. Interestingly, these factors have also been associated in other studies with the development of mental disorders in professionals, such as burnout, anxiety, and depression, which are the main causes of leave and early departure from work^(17,19,20)^.

To overcome the challenges identified, the participants in this study reflected on the importance of the topic of death being developed in a cross-cutting manner in health education, enabling students to identify and gradually develop the skills necessary to deal with it in an assisted manner. They also emphasized the importance of spaces for dialogue, welcoming mourning, and emotional release, both in the academic environment and at work, as well as the creation of multidisciplinary study groups that encourage an active stance in the healthcare process.

These reflections are echoed in a 2025 qualitative study with students and faculty members from a university in Brussels (Belgium) who participated in Compassionate Week, a festival about death, grief, and serious illness. Participants reported seeking out the festival as a way to deal with personal losses, support loved ones, and expand their knowledge on the subject. The event was perceived as a safe space for emotional expression and the sharing of narratives, contributing to a broader understanding of death and grief as inherent dimensions of life, including in the academic context, as well as promoting the normalization of dialogue about finitude and strengthening emotional support^(21)^.

Those needs are in line with a study that analyzed the presence of subjects related to education about death, loss, and grief in 103 medical and nursing courses in Brazil, which found that 83% of the courses address death in compulsory subjects, but only 28% include content related to education about death, loss, and grief, without ever treating it as a central theme. These data highlight the fragility and urgency of a reformulation of the education processes of the health professions, so that complex situations inherent to professional practice, such as death, are explored in depth in undergraduate courses, offering students the opportunity to understand the technical, symbolic, and emotional nuances involved^(7)^.

Furthermore, the relevance of detailed learning about coping with death was highlighted in a study conducted in England, which investigated the experience of parents whose babies died before, during, or after birth. The results showed that the terminology used by professionals to refer to the death of the baby significantly impacts the health of the parents: those informed that they were “losing their baby” instead of “having a miscarriage” showed greater preparedness to face labor, the birth of the stillborn baby, and to see and hold it. According to the authors, this terminology validates the loss, facilitating the farewell and the grieving process^(22)^. These findings corroborate the statements in the subcategory “gradual and safe contact with the experience,” which emphasize the importance of Welcoming and the words used by professionals when conveying the news of a death.

Authors argue that contact with the end of life causes a breakdown in the ego defenses of students, teachers, and health professionals, forcing them to face emotions related to loss and detachment^(10,23)^. This encourages reflection on the inevitability of death and the assumption of difficulties in dealing with it, including beliefs, concepts, and prejudices. Continuous and assisted engagement with the topic normalizes death in academic and professional circles, facilitating the exchange of knowledge for better preparation^(7,9,22)^. The testimony of DO7, in the same subcategory, reinforces this perspective by stating that the supervised confrontation with death during academic education enabled the development of their confidence and maturity to deal with these processes.

In Freire’s conception, listening and dialogue constitute the basis of human relations. There is no encounter without dialogue, just as there is no respect without listening. Through dialogue, contradictions and limits of the situations experienced emerge, revealing hidden factors that underpin interpretations of reality. Joint reflection allows individuals to know and transcend the limits of their own uniqueness, finding new ways of being and existing in the world^(24,25,26)^.

This logic applies to spaces dedicated to the expression of grief (mentioned in the third subcategory), which help students confront the emotional challenges of education, bringing benefits such as reduced stress, increased productivity and focus, improved empathy, and greater ability to deal with stressful situations such as death^(26)^. An example of these spaces are *Death Cafés*—an initiative that originated in the United Kingdom and is currently present in more than 90 countries—informal community gatherings for sharing experiences of loss and reflections on finitude. In these activities, participants can expand their emotional and practical knowledge about death and grief, in addition to strengthening community support networks, contributing to the promotion of more integrated social responses to the care of bereaving people^(27)^. In Brazil, however, the provision of this type of service in public universities remains centered in the Southeast Region and is intended exclusively for medical students^(26)^.

In this context, the creation of multidisciplinary study groups (fourth subcategory) is a promising idea, as it encourages the sharing of experiences among people with different knowledge and skills to overcome similar adversities, enabling collective growth and the development of solutions consistent with the reality of each context. Therefore, by opening themselves up to learning, professionals not only feel more prepared to face adverse situations, but also learn to identify risk factors to improve care, teaching quality, and the effectiveness of public policies^(7,8,9,17,22)^.

By taking a leading role in their learning process, health professionals develop awareness of their ability to feel, connect, and reflect, individually and collectively, on their rights and responsibilities. Even in the face of difficult or seemingly impossible circumstances, the investigative spirit, hope, and commitment to improving reality endure, as adversity comes to represent not the end, but the boundary between what is and what can be (last subcategory). Thus, by constructively discussing life’s problems, individuals transcend limitations and make the “unprecedented feasible” a possible reality^(1,24,25)^.

In this way, a formative process centered on loving dialogue and respectful relationships between educators and students creates a welcoming environment, capable of promoting joint growth and enabling those involved to critically understand their reality and actively transform it. In this sense, the pursuit of knowledge from undergraduate studies facilitates the awareness of future professionals about the importance of continuing education and the commitment to continuous evaluation of practice through the action-reflection-action cycle^(24,25)^.

### Advances in the Health Field and Limitations of the Study

The study contributes to the health field by highlighting the complexity of dealing with death in the context of birth, highlighting the need for comprehensive education that addresses technical, emotional, and symbolic aspects. The importance of welcoming spaces, dialogue, and multidisciplinary groups for the exchange of knowledge is emphasized, strengthening the preparation of professionals and supporting policies that promote the mental health and well-being of workers and bereaved families.

Limitations include the pandemic context, which hindered greater participation, and the restricted scope of the reality investigated. Thus, future research on the topic is recommended in other education contexts, in different periods, and with different methodological designs, in order to enable comparison. We also emphasize the importance of the participation of men in the health field in investigations involving sensitive topics related to women’s health, considering that, in this research, only women felt compelled to participate.

## CONCLUSION

The objective of this study was to identify solutions to prepare health professionals to deal with death in the context of birth, based on the experiences of teachers and students. The results point to the need for health education to consider the symbolic and emotional specificities surrounding death in this scenario, promoting the cross-cutting inclusion of the theme in the curriculum. We advocate gradual and assisted contact with the theme, linked to the creation of spaces for listening and the formation of multidisciplinary study groups, in order to encourage welcoming of different types of grief and strengthen professional leadership. Dialogue between different areas of knowledge proved to be fundamental in recognizing weaknesses and proposing strategies compatible with each education reality. In this process, Freire’s framework proved to be an important ally, supporting the need for listening, dialogue, and action-reflection-action as foundations for the construction of more sensitive, critical, and transformative practices in addressing death in health professional education.

## DATA AVAILABILITY

The entire dataset supporting the results of this study was published in the article itself.
