# Supplementary material for: Confronting death at birth: dialogues and solutions from health teachers and students
Source: Rev Esc Enferm USP. 2026 Apr 10;60:e20250381. doi: 10.1590/1980-220X-REEUSP-2025-0381en (PMC13090035; doi:10.1590/1980-220X-REEUSP-2025-0381en)
Supplement: Translation

# Translation: Enfrentamento da morte no nascimento: diálogos e soluções de docentes e discentes da saúde

**DOI:** 10.1590/1980-220X-REEUSP-2025-0381pt

**Authors:** Isadora Ferrante Boscoli de Oliveira Alves, Margarete Maria de Lima, Juanah Oliveira Debetio, Kênia Lara Silva, Maria Ligia dos Reis Bellaguarda, Roberta Costa

**Affiliations:** 1 Universidade Federal de Santa Catarina, Programa de Pós-graduação em Enfermagem, Florianópolis, SC, Brasil.; 2 Universidade Federal de Minas Gerais, Escola de Enfermagem, Belo Horizonte, MG, Brasil.

**Keywords:** Educação Interprofissional, Docentes, Estudantes, Atitude Frente a Morte, Obstetrícia

## Abstract

**Objetivo:**

Identificar soluções para preparar os profissionais de saúde para o enfrentamento da morte no contexto do nascimento a partir da vivência de docentes e discentes.

**Método:**

Estudo qualitativo, problematizador, realizado com 10 docentes e 2 discentes dos cursos de medicina, enfermagem e psicologia de uma universidade do sul do Brasil. A coleta de dados ocorreu por meio de grupos focais, organizados nas etapas do arco da problematização de Charles Maguerez. A análise seguiu a operacionalização de Minayo e o referencial teórico de Paulo Freire.

**Resultados:**

Profissionais da saúde relataram dificuldades no acolhimento aos distintos tipos de luto, associadas a lacunas formativas, limitações institucionais e ausência de espaços de escuta. Como soluções, destacaram a inserção transversal da temática nas graduações, a criação de espaços de apoio emocional, o incentivo a grupos interdisciplinares e o fortalecimento do protagonismo profissional diante de desafios éticos e assistenciais.

**Conclusão:**

O compartilhamento de experiências revelou potencial formativo e transformador, evidenciando a urgência de mudanças curriculares e institucionais para qualificar o cuidado.

## INTRODUÇÃO

A morte marca o fim da vida e caracteriza-se como um evento biológico e social, capaz de evocar diferentes significados e reações emocionais em quem a enfrenta. Embora seja parte do ciclo da vida, a morte é frequentemente temida pela maioria das pessoas, especialmente quando ocorre de maneira violenta ou abrupta. Nesse sentido, mortes inesperadas, como em um contexto de nascimento, costumam provocar reações de espanto, revolta e profundo sofrimento nas pessoas que a vivenciam^(1,2,3)^.

Segundo dados da Organização Pan-americana de Saúde (OPAS), todos os dias aproximadamente 830 mulheres morrem de causas evitáveis relacionadas à gravidez e ao parto em todo o mundo, sendo 99% desses óbitos em países em desenvolvimento. No Brasil, essas taxas aumentaram em 94,4% durante a pandemia da Covid-19, chegando a 107,53 casos para cada 100 mil Nascidos Vivos (NV) em 2021^(4)^. Já para o óbito neonatal, que corresponde a morte entre os primeiros 28 dias de vida de um bebê, estima-se uma taxa de aproximadamente 17 óbitos para cada 1.000/NV no ano de 2019, o equivalente a 6.700 mortes por dia. No Brasil, a média registrada foi de 12,4 óbitos/1.000 NV em 2018, um avanço comparado com a década de 1940, em que as taxas chegaram a 146,6 óbitos/1.000 NV^(5,6)^. Nota-se, portanto, que casos de morte envolvendo o contexto do nascimento são comuns em todo o mundo e exigem atenção e preparo para o seu enfrentamento.

Autores destacam que a sociedade frequentemente atribui a figura do médico a responsabilidade de impedir que a morte aconteça. Assim, é comum que as formações em saúde se concentrem em desenvolver no futuro profissional habilidades técnicas para a realização de diagnóstico e intervenções clínicas, por vezes negligenciando aspectos psicossociais envolvidos na prática e no lidar com o paciente. Consequentemente, ao se depararem com experiências subjetivas, como as envolvidas no enfrentamento de uma morte, esses profissionais se veem sem as ferramentas e habilidades necessárias para lidar com a situação^(7,8,9)^.

Estudos indicam que profissionais despreparados ou com pouca experiência tendem a adotar atitudes negativas ao se depararem com uma situação de morte, no intuito de evitar as próprias emoções. Assim, esses profissionais assumem postura mecanizada e de aparente frieza no trato com os pacientes e seus familiares, criando uma relação distanciada e sem vínculo que contradiz os princípios do Sistema Único de Saúde (SUS) (universalidade, integralidade e equidade) e a Política Nacional de Humanização. Por outro lado, profissionais devidamente capacitados demonstram maior habilidade para administrar suas emoções e oferecer suporte às famílias, ajudando-as a superarem a perda de forma mais saudável^(2,8,9,10,11)^.

Elisabeth Kübler-Ross (1926–2004), pioneira dos cuidados paliativos, afirmava a importância de os profissionais de saúde conhecerem sua postura frente à morte antes de prestarem atendimento aos familiares, destacando o impacto que suas ações causam na superação do luto dos pacientes. A psiquiatra ressaltava a necessidade dos cursos de formação desenvolverem nos discentes a conscientização sobre a realidade da própria morte, no intuito de fazê-los refletirem sobre sua finitude e desmistificar o tema no seu fazer profissional^(1)^.

Frente a isso, pesquisas demonstram que os cursos de graduação da área da saúde integram conteúdos relacionados à morte em seus currículos. No entanto, tendem a uma abordagem técnica, focada em procedimentos assépticos, preenchimento de documentação de óbito e na conduta que os profissionais devem ter no momento do óbito. Dessa forma, não preparam os acadêmicos para conduzir essas situações de forma acolhedora, empática e humana, repercutindo negativamente na experiência dos familiares e no exercício da prática profissional^(8,9,12,13)^.

Logo, partindo do pressuposto de que os hospitais se tornaram locais primordiais para o nascimento; que os profissionais da saúde se deparam regularmente com o processo de morte nesse contexto e não dispõem de preparo técnico e emocional para lidar com essa situação; e de que suas ações repercutem diretamente na vida das famílias, tem-se como objetivo: identificar soluções para preparar os profissionais de saúde para o enfrentamento da morte no contexto do nascimento a partir da vivência de docentes e discentes.

## MÉTODO

### Desenho do Estudo

Trata-se de um estudo qualitativo, de abordagem problematizadora, fundamentado no referencial teórico de Paulo Freire^(14)^. A pesquisa faz parte de um macroprojeto de natureza mista-transformativa, intitulado “Proposta pedagógica para o enfrentamento da morte e do luto no contexto do nascimento na formação de profissionais da área da saúde”, que teve como objetivo a construção coletiva de uma proposta educativa voltada ao preparo de profissionais da área da saúde para lidar com a morte e o luto no contexto do nascimento. O relatório final do estudo foi elaborado de acordo com os Critérios Consolidados de Comunicação de Estudos Qualitativos (COREQ): 32 *checklist*.

### Local

Por buscar construir soluções para preparar os profissionais de saúde para o enfrentamento da morte no contexto do nascimento, a partir da vivência de docentes e discentes, os cenários escolhidos para esta investigação foram os cursos de graduação da área da saúde de uma universidade pública localizada no sul do Brasil. A escolha considerou os mais de 60 anos de história da instituição e sua classificação entre as cinco melhores universidades do país no Índice Geral de Cursos (IGC) de 2019, que a posicionou entre as instituições de ensino com padrão de excelência na classificação do MEC.

Embora a instituição ofereça vários cursos de graduação relacionados à área da saúde, para este estudo foram considerados apenas os de Enfermagem, Medicina e Psicologia, devido à sua atuação expressiva no contexto do nascimento e por lidarem diretamente com o processo de enfrentamento da morte e do luto das famílias nesse cenário. Essas três áreas também se destacam como as principais responsáveis pela produção científica sobre o tema, conforme apontado pelos achados da revisão de literatura.

### População, Critérios de Seleção e Definição da Amostra

A população investigada consistiu em um total de 264 discentes matriculados nas duas últimas fases dos cursos de graduação em Enfermagem, Medicina e Psicologia da universidade mencionada (58 de Enfermagem, 134 de Medicina e 72 de Psicologia). No entanto, apenas 38 discentes foram incluídos neste estudo, por terem participado de uma etapa anterior do macroprojeto, que consistiu no preenchimento de um questionário relacionado à abordagem e o preparo recebido na formação para lidar com situações de morte e luto. Também foram considerados 41 docentes mencionados por esses discentes como potenciais participantes nesse questionário, por ministrarem disciplinas ou desenvolverem pesquisas sobre saúde materno-infantil e/ou os processos de morte e luto. Os convites foram realizados por meio do e-mail institucional e incluíram: uma carta de apresentação do estudo, o Termo de Consentimento Livre e Esclarecido (TCLE) e uma ficha de caracterização.

Como critérios de elegibilidade, adotaram-se: ser docente e ministrar aulas para os cursos de graduação em Enfermagem, Medicina ou Psicologia da universidade selecionada, com pelo menos um ano de experiência; ou ser estudante de graduação matriculado nas duas últimas fases dos cursos mencionados e ter participado da primeira fase do macroprojeto. Foram excluídos os docentes temporários.

### Coleta de Dados

A coleta de dados foi realizada por meio de grupos focais, conduzidos por uma animadora — pesquisadora principal, na época doutoranda em enfermagem, com experiência em pesquisas qualitativas, responsável por apresentar o tema e guiar as discussões de forma ativa e participativa —, e por uma relatora — também doutoranda em enfermagem, com experiência em entrevistas e atividades em grupo, que observou e registrou em diário de campo dados relativos à interação dos participantes, de maneira não participativa —, além dos próprios participantes^(14)^. Por pertencerem à instituição pesquisada, tanto a animadora quanto a relatora já conheciam a maioria dos participantes, embora de forma superficial.

Para adequar a pesquisa à disponibilidade dos convidados, foram organizados três momentos de coleta: um encontro virtual, um presencial e uma etapa de validação. Tanto a dinâmica do encontro virtual quanto a do presencial foi repetida três vezes, em datas distintas, totalizando seis grupos focais (três *online* e três presenciais). Para tanto, utilizou-se um roteiro adaptado do arco da problematização de Charles Maguerez, visando garantir o rigor metodológico da pesquisa. O roteiro seguiu as seguintes etapas: 1) Reconhecimento da realidade; 2) Listagem dos pontos-chave; 3) Teorização; 4) Hipóteses de solução; e 5) Aplicação à realidade. Ressalta-se que cada participante compareceu apenas uma vez em cada etapa da coleta^(15)^.

O encontro virtual foi realizado por meio da plataforma *Google Meet* ^®^. No dia previsto, os participantes foram recepcionados pela animadora e pela relatora, que fizeram uma breve apresentação de suas credenciais, dos objetivos da pesquisa e das diretrizes para condução do grupo naquele dia. Em seguida, os participantes foram informados sobre a gravação do encontro, a qual teve início somente após o consentimento de todos.

Para iniciar a fase de desenvolvimento, foi realizada uma dinâmica de “quebra-gelo”, com base em três perguntas disparadoras: “a) O que representa a morte e como eu lido com ela no meu exercício profissional?; b) Como percebo a experiência da morte no contexto do nascimento?; e c) Como avalio o preparo dos profissionais da saúde para lidarem com a morte no contexto do nascimento?”. Em seguida, foi solicitado aos participantes que inserissem imagens e/ou palavras representativas de suas respostas no aplicativo *Jamboard* ^®^, dentro do prazo de 15 minutos. Ao final, cada integrante apresentou suas reflexões, dando início à primeira etapa do arco de Charles Maguerez (Reconhecimento da realidade).

A partir disso, os participantes identificaram os pontos de conflito envolvidos na questão (2ª etapa do arco); teorizaram sobre sua ocorrência (3ª etapa); e articularam ideias compatíveis com sua realidade de atuação para solucioná-los (4ª e 5ª etapas). Os elementos trazidos pelos participantes em cada etapa foram registrados em forma de tópicos no próprio aplicativo, permitindo o acompanhamento simultâneo e a ciência de todos. Ao final, a animadora agradeceu a participação e informou as datas do segundo encontro. As atividades no *Jamboard* ^®^ foram fotografadas e utilizadas, juntamente com as gravações, para a preparação do encontro presencial.

O segundo momento de interação do grupo foi realizado presencialmente na própria universidade, seguindo todas as medidas de segurança recomendadas pelo Ministério da Saúde para conter a disseminação da Covid-19, ainda vigente à época. A animadora novamente recepcionou os participantes e informou sobre a gravação. Em seguida, foi realizada uma nova dinâmica de “quebra-gelo”, na qual foram apresentadas as imagens das atividades desenvolvidas nos grupos virtuais no aplicativo *Jamboard* ^®^, seguidas de uma síntese dos principais temas discutidos. Os dados foram dispostos em um quadro para que os participantes pudessem revisar, corrigir, complementar ou excluir informações, até que fosse alcançado consenso entre todos e a saturação dos dados.

Como os encontros presenciais também ocorreram separadamente, a pesquisadora principal promoveu uma etapa adicional de validação dos conteúdos produzidos. Para isso, foi enviado aos participantes, por e-mail, um arquivo contendo os dados sistematizados dos dois momentos de coleta, para que pudessem validar e/ou propor alterações no conteúdo, no prazo de 15 dias úteis. Ao final, foram solicitadas apenas correções textuais, encerrando-se assim a fase de validação.

A média de duração de cada grupo, tanto *online* quanto presencial, foi de aproximadamente duas horas. Todas as gravações foram transcritas pela pesquisadora principal. A coleta ocorreu entre os meses de agosto de 2022 e março de 2023.

### Análise e Tratamento dos Dados

O tratamento dos dados seguiu a proposta operativa de Minayo^(14)^, onde foram mapeadas as determinantes fundamentais do campo e seus atores; interpretados os fatores empíricos observados, agrupando-os em categorias e realizada a análise por inflexão para apreender o conteúdo concreto e o abstrato dos discursos, a fim de compreender os sentidos que os permeiam. Em seguida, os dados, previamente organizados com base nas etapas do arco da problematização, foram analisados à luz do referencial teórico de Paulo Freire, resultando em categorias e subcategorias de sentido.

### Aspéctos Éticos

Para preservar o anonimato dos participantes, os nomes dos docentes foram substituídos pela sigla “DO” e os dos discentes por “DI”, seguidos de um número correspondente à ordem de participação (DI1, DO2, DI3). O estudo seguiu as diretrizes das Resoluções nº 466/2012 e nº 510/2016, do Conselho Nacional de Saúde, bem como as orientações da Comissão Nacional de Ética em Pesquisa (CONEP) e do Ofício nº 2/2021, que dispõe sobre os procedimentos de pesquisa em ambientes virtuais. A pesquisa foi submetida ao Comitê de Ética em Pesquisa da universidade e aprovada sob o parecer nº 5.287.016, tendo sido iniciada apenas após a assinatura do Termo de Consentimento Livre e Esclarecido (TCLE) pelos participantes.

## RESULTADOS

Foram convidados 41 docentes para participar do estudo, dos quais 10 efetivamente compareceram às etapas de coleta. Desses, oito pertenciam ao curso de Enfermagem, um de Medicina e um de Psicologia. Todas as participantes eram mulheres, com titulação de doutorado. No que se refere ao estado civil, três eram solteiras, duas divorciadas e as demais casadas. A média de tempo de atuação na universidade entre as docentes participantes foi de aproximadamente 8,2 anos.

No que diz respeito aos discentes, dos 38 contactados, 11 manifestaram interesse em seguir participando da pesquisa. No entanto, apenas duas estudantes puderam estar presentes nos encontros: uma do curso de Enfermagem e outra de Medicina. Ambas eram do sexo feminino, uma casada e a outra solteira, com idades entre 22 e 26 anos. Ao todo, a etapa de coleta de dados contou com a participação de 12 pessoas: 10 docentes e 2 discentes.

Considerando as fases do arco da problematização e o referencial de Paulo Freire, os dados do estudo foram organizados em duas categorias principais de sentido, subdivididas em quatro e cinco subcategorias, respectivamente: 1) Reconhecendo a realidade e seus desafios: a) O significado da morte no contexto do nascimento diverge de outros cenários; b) Acolhimento de lutos diferentes; c) Conhecimento especializado e disponibilidade emocional; d) Limitações institucionais. 2) Identificando hipóteses de solução viáveis: a) Tornando a morte um tema transversal; b) Contato gradual e seguro com a experiência; c) Espaços para a vazão de sentimentos; d) Grupos de estudo multidisciplinares; e) Estímulo ao envolvimento ativo: o profissional como agente de mudança.

### Reconhecendo a Realidade e Seus Desafios: O Significado da Morte no Contexto do Nascimento Diverge de Outros Cenários

Os dados indicam que a experiência da morte no contexto do nascimento impacta os profissionais de saúde de maneira singular, diferenciando-se de outras perdas. Isso se deve ao significado simbólico do início da vida, aliado à associação frequente com iniquidades sociais e falhas na assistência, o que atribui à morte neonatal/materna uma carga emocional específica.

*A morte no contexto do nascimento é algo quase desestabilizador [...] quando isso acontece em um contexto de centro obstétrico [...] e todo aquele espaço está preparado para a vida, e de repente traz uma situação de morte, é algo muito delicado e difícil as vezes de lidar [...] tanto que a grande maioria foge [...] ninguém quer ter essa incumbência”.* (DO2)

*A gente percebe que a morte no contexto do nascimento está diretamente relacionada com os indicadores sociais. Ela reflete a qualidade da assistência à saúde para essa população, as interseccionalidades, porque a gente sabe que morrem mais mulheres negras, indígenas, de classe baixa [...] e apesar de a gente ter as políticas públicas [...] a gente sabe que nós estamos muito longe de alcançar as metas, principalmente em relação à mortalidade materna [...] [que] são, na sua grande maioria, mortes evitáveis.* (DO6)

### Reconhecendo a Realidade e Seus Desafios: Acolhimento de Lutos Diferentes

O contexto do nascimento envolve diferentes tipos de luto, que variam conforme o momento da morte (início da gestação, final da gestação, pós-parto), o tipo de óbito (materno, fetal ou neonatal) e suas causas (espontâneas, previstas ou esperadas, erros na identificação precoce de agravos e manejo, entre outras). Além disso, o luto é experienciado por diferentes sujeitos (pais, mães, irmãos, avós, profissionais de saúde, entre outros) e está associado ao significado que o falecido tinha para cada membro da família.

*O pós-cureta a gente acaba não lidando [com a mulher] [..] Ela muitas vezes vai ficar internada um tempo antes do procedimento [...] vivendo esse luto, vivendo essa perda e ninguém tem esse olhar. É real, não tem uma pessoa que vai lá dar um apoio e um suporte porque: “aí, é bem no iniciozinho, a barriga nem cresceu.* (DO2)

*Uma vez eu acompanhei uma morte materna [...] e aí eu vi a irmãzinha chegar com dez anos e pedir para segurar [o bebê], e ela já se assumindo no papel de mãe, uma criança de dez anos. Ela também perdeu a mãe, sabe?.* (DO9)

*[...] a morte de um bebê é quando tu perdes um sonho.[...] Agora, quando a gente perde a mãe eu acho que é muito complicado, principalmente para o pai que ficou e se ficou ainda o bebê. [...] É como se fosse riscada uma história. Então dói de todas as formas, vai doer a morte de um idoso, de um bebê, de uma criança, de um adolescente, mas eu acho que são dores que tem conotações diferentes porque vão ter sonhos diferentes, envolvimentos diferentes, expectativas diferentes.* (DO11)

### Reconhecendo a Realidade e Seus Desafios: Conhecimento Especializado e Disponibilidade Emocional

A disponibilidade emocional exigida para o enfrentamento da morte e do sofrimento das famílias representa um desafio significativo para os profissionais, uma vez que demanda o manejo cuidadoso de sentimentos, experiências e crenças pessoais, bem como o domínio de competências técnicas específicas.

*Lidar com alguém que lida com a morte é saber silenciar a sua própria experiência de morte [...] Eu gostaria que uma pessoa me falasse que eu sou nova ainda* [para ter um filho]*, mas empatia não é sobre isso, empatia não é sobre fazer o que você queria que fizessem para você, empatia é fazer o que aquela pessoa precisa que seja feita com ela. Então entender essa dinâmica do contexto da pessoa silenciar e fazer isso é fundamental.* (DI3)

*Eu vejo que existem receitas básicas ou condutas protocoladas que a gente faz [...] Por exemplo, se você tem uma mãe que perdeu um bebê, você não coloca ela no alojamento junto com as outras mães [...]. Quando um bebê nasce* [morto]*, você sempre estimula que a mãe pegue aquele bebê e possa fazer um reconhecimento na despedida [...] Mas às vezes [...] a resposta dela é não. Aí você espera, dá um tempinho [...] Aí, muitas vezes, elas mudam de opinião [...]. Então assim, a gente tem formas de tentar amenizar essas dores [...] mas eu acho que os profissionais precisam olhar através, para tentar entender o que as pessoas estão vivenciando.* (DO9)

*A gente teve uma experiência muito pesada de uma mãe que sabia que o neném não sobreviveria muitas horas após a parto, e ela tinha outras crianças. Então as outras crianças, junto ao pai e a mãe, fizeram uma cartinha [...] então, quando ela ganhou o bebê, o bebê veio para nós, com vida, para a gente deixar ele partir. [...] A gente colocou toda a roupinha [...] e leu a carta para ele* [bebê] *[...]* [e] *ficou com ele no colo [...]. Então não é fácil,* [porque] *tu está dando tchau para uma criancinha que nem é tua!.* (DO11)

### Reconhecendo a Realidade e Seus Desafios: Limitações Institucionais

Para os participantes da pesquisa, a alta rotatividade de profissionais nas instituições de saúde, motivada pela baixa remuneração salarial, acarreta sobrecarga de trabalho, mecanização da assistência e fragilização do entrosamento entre as equipes. Consequentemente, esses fatores dificultam o aporte humanizado do luto, além de aumentar a frequência de mortes no ambiente de trabalho.

*Semana passada a gente estava conversando que a morte materna está aumentando as estatísticas e [...]* [isso] *não podia acontecer, porque a morte materna é um erro, é uma falha [...] E o que a gente vê? Tem muita mudança de equipe [...] porque o salário não compensa [...]* [o valor é] *um terço, em comparação a trabalhar no estado, ninguém fica.* (DO2)

*Eu atuo bastante na atenção primária e [lá] isso é algo mais [distante] ainda, porque parece que já tem que pular para outro assunto: “vamos encaminhar para o psicólogo, isso já não é mais da minha alçada”, sabe? É uma fuga [...] Então, no hospital, a gente já tem pouco preparo [...], [mas] eu acho que na atenção primária,* [é] *menos ainda.* (DO6)

*A gente escuta muito: “eu não tenho nem tempo de cuidar dos vivos, vou cuidar dos que morreram?”. Não que os que morreram não tenham importância, mas eu estou sem tempo. E aí eu acho que forma um ciclo vicioso [...] aí quando tem tempo eu já nem vou porque eu nem faço isso no andamento das coisas que eu preciso fazer.* (DO12)

### Identificando Hipótese de Solução Viáveis: Tornar a Morte um Tema Transversal

Considerando a abrangência do tema e o impacto que causa no fazer assistencial, os participantes concluíram que a temática da morte deveria ser tratada de forma transversal nas formações em saúde.

*Acho que a graduação precisa sempre ter, de forma transversal,* [e em] *todas as disciplinas, alguns conteúdos vinculados como a morte e o cuidar de pessoas e família, porque é algo que tem que ser constante. [...] Ter uma proposta transversal e que abordasse os vários tipos de morte, não é um curso de especialização, mas é o mínimo que o aluno tem que ter de conceito, de experiências, de estratégias pedagógicas, para ele ir amadurecendo.* (DO7)

*No contexto do nascimento, quando acontece ali o aborto [...] também existe o aborto ilegal. [...]* [E] *essas pessoas chegam para a gente nos serviços e também foram atravessadas por um processo de luto, de morte, de perda, de julgamento social [...] de saúde psicossocial. Então, assim, se eu pensar em trabalhar isso na formação, eu acho que é um tema bioético* [transversal] *que faz parte.* (DO8)

### Identificando Hipótese de Solução Viáveis: Contato Gradual e Seguro Com a Experiência

No quesito aprendizagem, foi ressaltada a importância de proporcionar um contato gradual e realista com a temática da morte, para que os discentes compreendam sua complexidade e desenvolvam as habilidades necessárias para enfrentá-la.

*Eu vi crianças nascendo sem cérebro [...],vi mães que sabiam que a criança já estava falecida e estavam esperando a hora do parto para parir [...] e isso eu vivi na graduação. Para mim, qual foi o meu benefício? Tinha duas enfermeiras obstétricas, que me acompanhavam na época do estágio [...]* [e] *elas me deram todo o suporte, além de emocional, a questão técnica, eu acompanhava elas, fazia toda abordagem junto com elas. Então isso foi muito importante e me trouxe também maturidade profissional.* (DO7)

*Todo mundo lembra do primeiro óbito que acompanhou [...] e é isso que vai nos trazer segurança, nos trazer conhecimento e reflexão. [...] Quem já perdeu lembra que as falas realmente ficam na sua memória [...], o que foi bom [...] e as falas que* [nos] *fizeram desmoronar. Então você tem que ter muito cuidado quando você fala.* (DO9)

### Identificando Hipótese de Solução Viáveis: Espaços para a Vazão de Sentimentos

Destacou-se a importância dos grupos de apoio, nos quais docentes e discentes possam expressar seus sentimentos relacionados ao luto, compartilhar experiências e participar de atividades conjuntas de educação continuada, visando ao desenvolvimento de competências técnicas e sensíveis para o manejo adequado de situações envolvendo morte e luto.

*A instituição, considerando recursos, é muito difícil ela ter um profissional que é vinculado a ti. Psicólogo não vai poder te dar o suporte porque é contra os próprios princípios ter laços de profissionais ali.* [Buscar] *um profissional fora, a maioria não tem dinheiro para pagar. E a instituição também não tem. Então, o que é que eu sempre vi? Tu cria teus pequenos grupos de discussão sobre os problemas vividos [...] para dar subsídio tanto teórico quanto de troca de experiência para novos enfrentamentos. Mas a grande maioria dos grupos é sempre assim: “não tenho tempo, não tenho tempo.* (DO7)

*Eu fiquei muito admirada em descobrir através da minha tese [...] que os profissionais de modo geral têm muita dificuldade em lidar com a morte, principalmente para dar a notícia [...] para os familiares. [...] daí eu percebi [...] que os profissionais sofrem muito [...]. E quando essas mortes são constantes no teu trabalho, o sofrimento é maior ainda. [...] Então assim, eu acho que tem que ter esse acompanhamento para os profissionais que também não tem momentos de reflexão* (DO10)

### Identificando Hipótese de Solução Viáveis: Grupos de Estudo Multidisciplinares

Foi também sugerida a realização de momentos de troca multidisciplinares nas universidades, com o objetivo de promover o entendimento das diferentes perspectivas acerca da morte e do seu enfrentamento, favorecendo a superação de eventuais dificuldades inerentes a cada área de atuação.

*Eu acho que o apoio dos próprios colegas, pelo menos no nosso departamento [...] tu tens um grupo de docentes, que tem reuniões constantes [...] todo mundo tem doutorado, tem pós--doutorado, muitos cursos... Eu vejo uma troca muito saudável de experiências, de planejamentos, de como abordar o aluno. Pode não te dar um suporte 100%, mas eu entendo, no meu ponto de vista, que é saudável e ajuda bastante.* (DO7)

*Essa roda de conversa hoje aqui, que coisa boa até para nós que estamos participando [...]. Isso deveria acontecer mais vezes, nós precisamos falar mais desse assunto na sociedade de uma forma geral, também na formação e na assistência.* (DO10)

### Identificando Hipótese de Solução Viáveis: Estímulo ao Envolvimento Ativo: O Profissional Como Agente de Mudança

Duas participantes ressaltaram a importância de promover o “agenciamento” do profissional de saúde no processo formativo, incentivando-o a assumir postura proativa diante dos conflitos entre o treinamento recebido, as necessidades da população atendida e as normativas institucionais vigentes.

*Algo que eu acrescentaria seria entender as limitações institucionais [...] “Não pode entrar”, “não pode ver”, ‘’não pode levar’’ [...] tudo bem que a família inteira não possa ver uma criança que está na UTI [...] agora, será que está tudo bem a gente ter que mandar uma criança lá para o outro lugar [...] e tirá-la daquele berço pequeno? [...] Acho que entender as limitações institucionais nesses termos [...] é uma coisa importante [...]* [para] *perceber-se como potencial transformador dessas regras, sabe?.* (DI3)

*A gente é formado para trabalhar de um jeito legal e vem as regras da instituição [...]. Você paralisa, porque é um bom moço. Você aprendeu a seguir regras, não só porque elas são necessárias, mas porque elas falam também do nosso contrato social de se manter numa prática que seja coesa, não é? [...] Agenciamento significa justamente você ir criando as regras numa perspectiva [...] de dialogar com essas regras e agenciar um novo modo de vida, um novo modo de cuidar.* (DO5)

## DISCUSSÃO

Observa-se que docentes e discentes vivenciam situações de morte no contexto do nascimento ao longo da formação. Contudo, em razão da associação desse cenário ao início da vida, o significado da morte adquire conotações específicas que influenciam o enfrentamento tanto dos profissionais quanto dos enlutados. Para tanto, o acolhimento ao luto neste contexto apresenta complexidades, por envolver fatores simbólicos, crenças e iniquidades sociais frequentemente negligenciadas pelos processos formativos, resultando em preparo insuficiente e disponibilidade emocional limitada para conduzir adequadamente o processo de perda conforme as necessidades singulares de cada enlutado (depoimentos da primeira e segunda subcategoria).

Estudos salientam que a perda perinatal, frequentemente, não é reconhecida pela sociedade, já que os pais não têm a oportunidade de conviver com o filho por muito tempo. O fato fomenta no imaginário coletivo a crença de que o sofrimento é menor nesses casos e de que os pais não precisam de cuidados específicos^(8,9,11)^. Consequentemente, os pais em processo de perda são tolhidos de demonstrar seu pesar em público, ao mesmo tempo em que têm sua maternidade/paternidade invalidada pelo meio, inclusive pelos profissionais da saúde.

A acolhida eficaz requer práticas fundamentadas no diálogo e na construção de vínculos. Uma cartilha de educação para a morte, sugere que os profissionais de saúde precisam considerar seis elementos para compreender o processo de luto de uma pessoa: 1) quem era o falecido na vida do enlutado e o que ele representava; 2) a natureza do vínculo emocional entre eles; 3) as circunstâncias da morte e a forma como o enlutado recebeu a notícia; 4) histórico de luto complicado anteriores ao vivido; 5) a personalidade do enlutado; 6) e os fatores sociais envolvidos, como as influências culturais, étnicas, religiosas e a presença de rede de apoio^(16)^. Portanto, deixa explicita a importância dos profissionais desenvolverem habilidades de “silêncio e empatia” para ouvirem a dor do outro, de modo a se tornarem capazes de “olhar através das circunstâncias” e realizar uma assistência para além dos protocolos institucionais pré-estabelecidos (falas da terceira subcategoria).

A realização de práticas de cuidado complexas, como o acolhimento ao luto, demanda tempo, treinamento e a articulação das equipes de saúde. Consequentemente, tais práticas são diretamente afetadas quando há sobrecarga de trabalho, rotatividade profissional e mecanização da assistência (fatores mencionados na quarta subcategoria). Um estudo descritivo realizado em 35 unidades de saúde da família em um estado de médio porte do Brasil identificou que a alta rotatividade dos profissionais e a falta de capacitação especializada impactam os serviços prestados pelas equipes, resultando em avaliações insatisfatórias pelos usuários em quase todos os itens incluídos no *Primary Care Assessment Tool* (PCATool-Brasil)^(17)^.

Outro estudo^(18)^, realizado com 166 enfermeiros, indicou que a rotatividade nas instituições de saúde está relacionada a cinco fatores: apoio da chefia, recompensa, conforto físico, controle/pressão e coesão da equipe. Os dados apontam que o aumento da “recompensa” é o fator mais eficaz para reduzir a intenção de rotatividade, enquanto a diminuição do conforto físico e a baixa coesão da equipe a aumentam, o que é coerente com os depoimentos da subcategoria “limitações institucionais”. Curiosamente, esses fatores também foram associados em outras pesquisas ao desenvolvimento de distúrbios psíquicos nos profissionais, como *burnout*, ansiedade e depressão, principais causas de afastamentos e saídas precoces do trabalho^(17,19,20)^.

Para superar os desafios identificados, os participantes do presente estudo refletiram sobre a importância do tema da morte ser desenvolvidos de forma transversal nas formações em saúde, possibilitando que os discentes identifiquem e desenvolvam gradativamente as habilidades necessárias para enfrentá-la de maneira assistida. Ressaltaram ainda a relevância da existência de espaços para diálogo, acolhimento ao luto e vazão dos sentimentos, tanto no ambiente acadêmico quanto no trabalho, além da criação de grupos de estudo multidisciplinares que estimulem uma postura ativa no processo de cuidado em saúde.

Essas reflexões encontram eco em um estudo qualitativo de 2025 com estudantes e membros do corpo universitário de uma universidade em Bruxelas (Bélgica), que participaram da *Compassionate Week*, um festival sobre morte, luto e doença grave. Os participantes relataram buscar o festival como forma de lidar com perdas pessoais, apoiar pessoas próximas e ampliar seus conhecimentos sobre o tema. O evento foi percebido como um espaço seguro para a expressão emocional e o compartilhamento de narrativas, contribuindo para ampliar a compreensão da morte e do luto como dimensões inerentes à vida, inclusive no contexto acadêmico, além de favorecer a normalização do diálogo sobre a finitude e o fortalecimento do apoio emocional^(21)^.

Essas necessidades alinham-se a um estudo que analisou a presença de disciplinas relacionadas à educação para morte, perda e luto em 103 cursos de medicina e enfermagem no Brasil, o qual identificou que 83% dos cursos abordam a morte em disciplinas obrigatórias, mas apenas 28% contemplam conteúdos relacionados à educação para morte, perda e luto, sem jamais tratá-la como tema central. Esses dados evidenciam a fragilidade e a urgência de uma reformulação nos processos formativos das profissões da saúde, para que situações complexas e inerentes ao exercício profissional, como a morte, sejam aprofundadas nas graduações, oferecendo aos discentes a oportunidade de compreender as nuances técnicas, simbólicas e emocionais envolvidas^(7)^.

Ademais, a relevância de um aprendizado detalhado sobre o enfrentamento da morte foi evidenciada em estudo realizado na Inglaterra, que investigou a experiência de pais cujos bebês morreram antes, durante ou após o nascimento. Os resultados demonstraram que a terminologia utilizada pelos profissionais para se referirem à morte do bebê impacta significativamente a saúde dos pais: aqueles informados de que estavam “perdendo seu bebê”, em vez de “tendo um aborto espontâneo”, demonstravam maior preparo para enfrentar o trabalho de parto, o nascimento do natimorto e para vê-lo e segurá-lo. Segundo os autores, essa terminologia valida a perda, facilitando a despedida e a elaboração do luto^(22)^. Esses achados corroboram as falas da subcategoria “contato gradual e seguro com a experiência”, que ressaltam a importância do acolhimento e das palavras utilizadas pelos profissionais no momento de transmitir a notícia de um óbito.

Autores defendem que o contato com a terminalidade da vida provoca uma quebra nas defesas do ego de discentes, docentes e profissionais da saúde, obrigando-os a enfrentar as emoções relacionadas à perda e ao desapego^(10,23)^. Isso propicia reflexão sobre a inevitabilidade da morte e a assunção das dificuldades para lidar com ela, incluindo crenças, conceitos e preconceitos. A aproximação contínua e assistida ao tema naturaliza a morte no meio acadêmico e profissional, facilitando o intercâmbio de saberes para um melhor preparo^(7,9,22)^. O depoimento de DO7, na mesma subcategoria, reitera essa perspectiva ao afirmar que o enfrentamento supervisionado da morte, durante a formação acadêmica, possibilitou o desenvolvimento de sua confiança e maturidade para lidar com esses processos.

Na concepção freireana, escuta e diálogo constituem a base das relações humanas. Não há encontro sem diálogo, assim como não há respeito sem escuta. Por meio da dialogicidade emergem contradições e limites das situações vivenciadas, revelando fatores ocultos que sustentam as interpretações da realidade. A reflexão conjunta permite que os indivíduos conheçam e transcendam os limites da própria singularidade, encontrando novas formas de ser e estar no mundo^(24,25,26)^.

Essa lógica se aplica aos espaços destinados à expressão do luto (mencionados na terceira subcategoria), que auxiliam os discentes a confrontar desafios emocionais da formação, trazendo benefícios como redução do estresse, aumento da produtividade e do foco, melhoria da empatia e maior habilidade para lidar com situações estressantes, como a morte^(26)^. Um exemplo desses espaços são os *Death Cafés* — iniciativa originada no Reino Unido e atualmente presente em mais de 90 países —, encontros comunitários informais destinados ao compartilhamento de experiências de perda e reflexões sobre a finitude. Nessas atividades, os participantes podem ampliar seu conhecimento emocional e prático sobre morte e luto, além de fortalecer redes comunitárias de apoio, contribuindo para a promoção de respostas sociais mais integradas ao cuidado de pessoas enlutadas^(27)^. No Brasil, entretanto, a oferta desse tipo de serviço nas universidades públicas permanece centrado na Região Sudeste e é destinada exclusivamente a discentes de medicina^(26)^.

Nesse contexto, a criação de grupos de estudo multidisciplinares (quarta subcategoria) é uma ideia promissora, pois estimula a partilha de experiências entre pessoas com diferentes conhecimentos e habilidades para superar adversidades semelhantes, possibilitando crescimento coletivo e desenvolvimento de soluções condizentes com a realidade de cada contexto. Portanto, ao se abrirem ao aprendizado, os profissionais não apenas se sentem mais preparados para enfrentar situações adversas, mas também aprendem a identificar fatores de risco para aprimorar a assistência, a qualidade do ensino e a eficácia das políticas públicas^(7,8,9,17,22)^.

Ao assumirem protagonismo em seu processo de aprendizado, os profissionais da saúde desenvolvem consciência sobre sua capacidade de sentir, conectar-se e refletir, individual e coletivamente, sobre seus direitos e responsabilidades. Mesmo diante de circunstâncias difíceis ou aparentemente impossíveis, o espírito investigativo, a esperança e o compromisso com a melhoria da realidade perduram, pois a adversidade passa a representar não o fim, mas a fronteira entre o que é e o que pode vir a ser (última subcategoria). Assim, ao dialogar construtivamente sobre os problemas da vida, os indivíduos transcendem limitações e tornam o “inédito viável” uma realidade possível^(1,24,25)^.

Dessa forma, um processo formativo centrado no diálogo amoroso e na relação respeitosa entre educadores e educandos cria um ambiente acolhedor, capaz de promover crescimento conjunto e capacitar os envolvidos a compreender criticamente sua realidade e transformá-la ativamente. Nesse sentido, o exercício da busca pelo conhecimento desde a graduação facilita a conscientização dos futuros profissionais sobre a importância da educação permanente e o compromisso com a avaliação contínua da prática por meio do ciclo ação-reflexão-ação^(24,25)^.

### Avanços para a Área da Saúde e Limitações do Estudo

O estudo contribui para a área da saúde ao destacar a complexidade do enfrentamento da morte no contexto do nascimento, evidenciando a necessidade de uma formação integral que aborde aspectos técnicos, emocionais e simbólicos. Ressalta-se a importância de espaços de acolhimento, diálogo e grupos multidisciplinares para a troca de saberes, fortalecendo a preparação dos profissionais e subsidiando políticas que promovam a saúde mental e o bem-estar dos trabalhadores e famílias enlutadas.

Como limitações, destacam-se o contexto pandêmico, que dificultou maior adesão dos participantes, e o recorte restrito da realidade investigada. Assim, recomenda-se a realização de pesquisas futuras sobre o tema em outros contextos formativos, em diferentes períodos e com distintos desenhos metodológicos, de modo a possibilitar a comparação. Reforça-se, ainda, a importância da participação de homens da área da saúde em investigações que envolvam temas sensíveis e relacionados à saúde da mulher, considerando que, nesta pesquisa, apenas mulheres se sentiram impelidas a participar.

## CONCLUSÃO

O objetivo deste estudo foi identificar soluções para preparar profissionais de saúde para o enfrentamento da morte no contexto do nascimento, com base nas vivências de docentes e discentes. Os resultados apontam a necessidade de que a formação em profissões de saúde considerem as especificidades simbólicas e emocionais que envolvem o óbito nesse cenário, promovendo a inclusão transversal da temática nas grades curriculares. Defende-se um contato gradual e assistido com o tema, articulado à criação de espaços de escuta e à formação de grupos de estudo multidisciplinares, a fim de favorecer o acolhimento aos diversos tipos de luto e fortalecer o protagonismo profissional. O diálogo entre diferentes saberes revelou-se fundamental para reconhecer fragilidades e propor estratégias compatíveis com cada realidade formativa. Nesse processo, o referencial freireano mostrou-se um importante aliado, ao sustentar a necessidade de escuta, diálogo e ação-reflexão-ação como fundamentos para a construção de práticas mais sensíveis, críticas e transformadoras na abordagem da morte na formação em profissões de saúde.

## DISPONIBILIDADE DE DADOS

Todo o conjunto de dados que dá suporte aos resultados deste estudo foi publicado no próprio artigo.

# Translation: Afrontar la muerte en el parto: diálogos y soluciones de docentes y estudiantes de salud

**DOI:** 10.1590/1980-220X-REEUSP-2025-0381es

**Authors:** Isadora Ferrante Boscoli de Oliveira Alves, Margarete Maria de Lima, Juanah Oliveira Debetio, Kênia Lara Silva, Maria Ligia dos Reis Bellaguarda, Roberta Costa

**Affiliations:** 1 Universidade Federal de Santa Catarina, Programa de Pós-graduação em Enfermagem, Florianópolis, SC, Brasil.; 2 Universidade Federal de Minas Gerais, Escola de Enfermagem, Belo Horizonte, MG, Brasil.

**Keywords:** Educación interprofesional, Docentes, Estudiantes, Actitud Frente a la Muerte, Obstetricia

## Abstract

**Objetivo:**

Identificar soluciones para preparar a los profesionales de la salud para afrontar la muerte en el contexto del parto, basándose en las experiencias de profesores y estudiantes.

**Método:**

Se llevó a cabo un estudio cualitativo y problematizante con 10 profesores y 2 estudiantes de los cursos de medicina, enfermería y psicología de una universidad del sur de Brasil. La recolección de datos se llevó a cabo mediante grupos focales, organizados según las etapas del arco de problematización de Charles Maguerez. El análisis siguió la operacionalización de Minayo y el marco teórico de Paulo Freire.

**Resultados:**

Los profesionales de la salud informaron dificultades para aceptar diferentes tipos de duelo, asociadas con lagunas en la educación, limitaciones institucionales y falta de espacios para escuchar. Como soluciones, destacaron la inclusión transversal del tema en los cursos de licenciatura, la creación de espacios de apoyo emocional, el fomento de grupos interdisciplinarios y el fortalecimiento del liderazgo profesional ante los retos éticos y asistenciales.

**Conclusión:**

El intercambio de experiencias reveló el potencial educativo y transformador, destacando la urgencia de cambios curriculares e institucionales para mejorar la calidad de la atención.

## INTRODUCCIÓN

La muerte marca el final de la vida y se caracteriza como un evento biológico y social, capaz de evocar diferentes significados y reacciones emocionales en quienes la enfrentan. Aunque forma parte del ciclo de la vida, la muerte es a menudo temida por la mayoría de las personas, especialmente cuando ocurre de manera violenta o abrupta. En este sentido, las muertes inesperadas, como en un contexto de parto, suelen provocar reacciones de asombro, rebeldía y profundo dolor en las personas que las viven^(1,2,3)^.

Según datos de la Organización Panamericana de la Salud (OPS), cada día mueren aproximadamente 830 mujeres por causas evitables relacionadas con el embarazo y el parto en todo el mundo, y el 99 % de estas muertes se producen en países en desarrollo. En Brasil, estas tasas aumentaron un 94,4 % durante la pandemia de COVID-19, llegando a 107,53 casos por cada 100 000 nacidos vivos (NV) en 2021^(4)^. En cuanto a la mortalidad neonatal, que corresponde a la muerte entre los primeros 28 días de vida de un bebé, se estima una tasa de aproximadamente 17 muertes por cada 1000 NV en el año 2019, lo que equivale a 6700 muertes al día. En Brasil, la media registrada fue de 12,4 muertes/1000 NV en 2018, lo que supone un avance en comparación con la década de 1940, en la que las tasas alcanzaron las 146,6 muertes/1000 NV^(5,6)^. Por lo tanto, se observa que los casos de muerte relacionados con el contexto del nacimiento son comunes en todo el mundo y requieren atención y preparación para afrontarlos.

Los autores destacan que la sociedad suele atribuir al médico la responsabilidad de impedir que se produzca la muerte. Por lo tanto, es común que la formación en salud se centre en desarrollar en el futuro profesional habilidades técnicas para realizar diagnósticos e intervenciones clínicas, a veces descuidando los aspectos psicosociales involucrados en la práctica y en el trato con el paciente. En consecuencia, cuando se enfrentan a experiencias subjetivas, como las relacionadas con afrontar una muerte, estos profesionales se ven sin las herramientas y habilidades necesarias para lidiar con la situación^(7,8,9)^.

Los estudios indican que los profesionales sin preparación o con poca experiencia tienden a adoptar actitudes negativas cuando se enfrentan a una situación de muerte, con el fin de evitar sus propias emociones. Así, estos profesionales adoptan una postura mecanizada y de aparente frialdad en el trato con los pacientes y sus familiares, creando una relación distante y sin vínculo que contradice los principios del Sistema Único de Salud (SUS) (universalidad, integralidad y equidad) y la Política Nacional de Humanización. Por otro lado, los profesionales debidamente capacitados demuestran una mayor habilidad para manejar sus emociones y ofrecer apoyo a las familias, ayudándolas a superar la pérdida de una manera más saludable^(2,8,9,10,11)^.

Elisabeth Kübler-Ross (1926–2004), pionera de los cuidados paliativos, afirmaba la importancia de que los profesionales de la salud conozcan su postura frente a la muerte antes de atender a los familiares, destacando el impacto que sus acciones causan en la superación del duelo de los pacientes. La psiquiatra subrayaba la necesidad de que los cursos de formación desarrollaran en los estudiantes la conciencia sobre la realidad de la propia muerte, con el fin de hacerles reflexionar sobre su finitud y desmitificar el tema en su práctica profesional^(1)^.

Ante esto, las investigaciones demuestran que los cursos de licenciatura en el área de la salud integran contenidos relacionados con la muerte en sus planes de estudio. Sin embargo, tienden a adoptar un enfoque técnico, centrado en procedimientos asépticos, la cumplimentación de la documentación de defunción y la conducta que deben tener los profesionales en el momento de la muerte. De este modo, no preparan a los estudiantes para manejar estas situaciones de manera acogedora, empática y humana, lo que repercute negativamente en la experiencia de los familiares y en el ejercicio de la práctica profesional^(8,9,12,13)^.

Por lo tanto, partiendo de la premisa de que los hospitales se han convertido en lugares primordiales para el nacimiento; que los profesionales de la salud se enfrentan regularmente al proceso de la muerte en este contexto y no cuentan con la preparación técnica y emocional para lidiar con esta situación; y que sus acciones repercuten directamente en la vida de las familias, el objetivo es identificar soluciones para preparar a los profesionales de la salud para afrontar la muerte en el contexto del nacimiento a partir de la experiencia de profesores y alumnos.

## MÉTODO

### Diseño del Estudio

Se trata de un estudio cualitativo, con un enfoque problematizante, basado en el marco teórico de Paulo Freire^(14)^. La investigación forma parte de un macroproyecto de naturaleza mixta-transformadora, titulado “Propuesta pedagógica para afrontar la muerte y el duelo en el contexto del nacimiento en la formación de profesionales de la salud”, cuyo objetivo era la construcción colectiva de una propuesta educativa orientada a la preparación de profesionales de la salud para lidiar con la muerte y el duelo en el contexto del nacimiento. El informe final del estudio se elaboró de acuerdo con los Criterios Consolidados de Comunicación de Estudios Cualitativos (COREQ): 32 checklist.

### Lugar

Con el fin de buscar soluciones para preparar a los profesionales de la salud para afrontar la muerte en el contexto del nacimiento, a partir de la experiencia de profesores y alumnos, los escenarios elegidos para esta investigación fueron los cursos de grado en el área de la salud de una universidad pública situada en el sur de Brasil.

La elección tuvo en cuenta los más de 60 años de historia de la institución y su clasificación entre las cinco mejores universidades del país en el Índice General de Cursos (IGC) de 2019, que la situó entre las instituciones educativas con un nivel de excelencia en la clasificación del Ministerio de Educación (MEC).

Aunque la institución ofrece varios cursos de licenciatura relacionados con el área de la salud, para este estudio solo se consideraron los de Enfermería, Medicina y Psicología, debido a su importante actuación en el contexto del nacimiento y por tratar directamente con el proceso de afrontamiento de la muerte y el duelo de las familias en este escenario. Estas tres áreas también se destacan como las principales responsables de la producción científica sobre el tema, según lo señalado por los hallazgos de la revisión de la literatura.

### Población, Criterios de Selección y Definición de la Muestra

La población investigada consistió en un total de 264 estudiantes matriculados en las dos últimas fases de los cursos de licenciatura en Enfermería, Medicina y Psicología de la universidad mencionada (58 de Enfermería, 134 de Medicina y 72 de Psicología). Sin embargo, solo 38 estudiantes fueron incluidos en este estudio, ya que habían participado en una etapa anterior del macroproyecto, que consistió en completar un cuestionario relacionado con el enfoque y la preparación recibida en la formación para lidiar con situaciones de muerte y duelo. También se consideraron 41 profesores mencionados por estos estudiantes como posibles participantes en este cuestionario, por impartir disciplinas o desarrollar investigaciones sobre salud materno-infantil y/o los procesos de muerte y duelo. Las invitaciones se realizaron a través del correo electrónico institucional e incluyeron: una carta de presentación del estudio, el Término de Consentimiento Libre y Esclarecido (TCLE) y una ficha de caracterización.

Como criterios de elegibilidad, se adoptaron los siguientes: ser docente e impartir clases en los cursos de licenciatura en Enfermería, Medicina o Psicología de la universidad seleccionada, con al menos un año de experiencia; o ser estudiante de licenciatura matriculado en las dos últimas fases de los cursos mencionados y haber participado en la primera fase del macroproyecto. Se excluyó a los profesores temporales.

### Recolección de Datos

La recolección de datos se realizó mediante grupos focales, dirigidos por una animadora —investigadora principal, en ese momento estudiante de doctorado en enfermería, con experiencia en investigaciones cualitativas, responsable de presentar el tema y guiar los debates de forma activa y participativa— y por una relatora —también estudiante de doctorado en enfermería, con experiencia en entrevistas y actividades en grupo, que observó y registró en un diario de campo datos relativos a la interacción de los participantes, de manera no participativa—, además de los propios participantes^(14)^. Al pertenecer a la institución investigada, tanto la animadora como la relatora ya conocían a la mayoría de los participantes, aunque de manera superficial.

Para adaptar la investigación a la disponibilidad de los invitados, se organizaron tres momentos de recolección: una reunión virtual, una presencial y una etapa de validación. Tanto la dinámica de la reunión virtual como la de la presencial se repitieron tres veces, en fechas diferentes, sumando un total de seis grupos focales (tres *en línea* y tres presenciales). Para ello, se utilizó un guion adaptado del arco de problematización de Charles Maguerez, con el fin de garantizar el rigor metodológico de la investigación. El guion siguió las siguientes etapas: 1) Reconocimiento de la realidad; 2) Enumeración de los puntos clave; 3) Teorización; 4) Hipótesis de solución; y 5) Aplicación a la realidad. Cabe destacar que cada participante asistió solo una vez a cada etapa de la Recolección^(15)^.

La reunión virtual se llevó a cabo a través de la plataforma Google Meet^®^. El día previsto, los participantes fueron recibidos por la animadora y la relatora, quienes hicieron una breve presentación de sus credenciales, los objetivos de la investigación y las directrices para dirigir al grupo ese día. A continuación, se informó a los participantes sobre la grabación de la reunión, que comenzó solo después de obtener el consentimiento de todos.

Para iniciar la fase de desarrollo, se realizó una dinámica para “romper el hielo”, basada en tres preguntas desencadenantes: “a) ¿Qué representa la muerte y cómo la afronto en mi ejercicio profesional?; b) ¿Cómo percibo la experiencia de la muerte en el contexto del nacimiento?; y c) ¿Cómo evalúo la preparación de los profesionales de la salud para afrontar la muerte en el contexto del nacimiento?”. A continuación, se pidió a los participantes que introdujeran imágenes y/o palabras representativas de sus respuestas en la aplicación Jamboard^®^, en un plazo de 15 minutos. Al final, cada miembro presentó sus reflexiones, dando inicio a la primera etapa del arco de Charles Maguerez (Reconocimiento de la realidad).

A partir de ahí, los participantes identificaron los puntos de conflicto relacionados con la cuestión (2.ª etapa del arco); teorizaron sobre su ocurrencia (3.ª etapa); y articularon ideas compatibles con su realidad de actuación para resolverlos (4.ª y 5.ª etapas). Los elementos aportados por los participantes en cada etapa se registraron en forma de temas en la propia aplicación, lo que permitió el seguimiento simultáneo y el conocimiento de todos. Al final, la animadora agradeció la participación e informó de las fechas de la segunda reunión. Las actividades en *Jamboard* ^®^ fueron fotografiadas y utilizadas, junto con las grabaciones, para la preparación de la reunión presencial.

El segundo momento de interacción del grupo se llevó a cabo de manera presencial en la propia universidad, siguiendo todas las medidas de seguridad recomendadas por el Ministerio de Salud para contener la propagación de la COVID-19, aún vigentes en ese momento. La animadora volvió a dar la bienvenida a los participantes y les informó sobre la grabación. A continuación, se llevó a cabo una nueva dinámica para “romper el hielo”, en la que se presentaron las imágenes de las actividades desarrolladas en los grupos virtuales en la aplicación Jamboard^®^, seguidas de un resumen de los principales temas discutidos. Los datos se dispusieron en un cuadro para que los participantes pudieran revisar, corregir, complementar o excluir información, hasta que se alcanzara el consenso entre todos y la saturación de los datos.

Dado que las reuniones presenciales también se celebraron por separado, la investigadora principal promovió una etapa adicional de validación de los contenidos producidos. Para ello, se envió a los participantes, por correo electrónico, un archivo con los datos sistematizados de los dos momentos de Recolección, para que pudieran validar y/o proponer cambios en el contenido, en un plazo de 15 días hábiles. Al final, solo se solicitaron correcciones textuales, con lo que se dio por concluida la fase de validación.

La duración media de cada grupo, tanto en línea como presencial, fue de aproximadamente dos horas. Todas las grabaciones fueron transcritas por la investigadora principal. La recolección se llevó a cabo entre los meses de agosto de 2022 y marzo de 2023.

### Análisis y Tratamiento de los Datos

El tratamiento de los datos siguió la propuesta operativa de Minayo^(14)^, en la que se mapearon los determinantes fundamentales del campo y sus actores; se interpretaron los factores empíricos observados, agrupándolos en categorías y se realizó el análisis por inflexión para captar el contenido concreto y abstracto de los discursos, con el fin de comprender los significados que los impregnan. A continuación, los datos, previamente organizados en función de las etapas del arco de problematización, se analizaron a la luz del marco teórico de Paulo Freire, lo que dio como resultado categorías y subcategorías de significado.

### Aspectos Éticos

Para preservar el anonimato de los participantes, los nombres de los docentes fueron sustituidos por la sigla “DO” y los de los estudiantes por “DI”, seguidos de un número correspondiente al orden de participación (DI1, DO2, DI3). El estudio siguió las directrices de las Resoluciones n.º 466/2012 y n.º 510/2016 del Consejo Nacional de Salud, así como las orientaciones de la Comisión Nacional de Ética en Investigación (CONEP) y la Oficina n.º 2/2021, que dispone sobre los procedimientos de investigación en entornos virtuales. La investigación fue sometida al Comité de Ética en Investigación de la universidad y aprobada mediante el dictamen n.º 5.287.016, habiéndose iniciado solo después de la firma del Término de Consentimiento Libre e Informado (TCLE) por parte de los participantes.

## RESULTADOS

Se invitó a 41 docentes a participar en el estudio, de los cuales 10 asistieron efectivamente a las etapas de Recolección. De ellos, ocho pertenecían al curso de Enfermería, uno al de Medicina y otro al de Psicología. Todas las participantes eran mujeres, con título de doctorado. En cuanto al estado civil, tres eran solteras, dos divorciadas y el resto casadas.

El promedio de tiempo de actuación en la universidad entre las docentes participantes fue de aproximadamente 8,2 años. En lo que respecta a los estudiantes, de los 38 contactados, 11 manifestaron interés en seguir participando en la investigación. Sin embargo, solo dos estudiantes pudieron asistir a las reuniones: una del curso de Enfermería y otra de Medicina. Ambas eran mujeres, una casada y la otra soltera, con edades comprendidas entre los 22 y los 26 años. En total, en la etapa de Recolección de datos participaron 12 personas: 10 docentes y 2 estudiantes.

Teniendo en cuenta las fases del arco de problematización y el marco de referencia de Paulo Freire, los datos del estudio se organizaron en dos categorías principales de significado, subdivididas en cuatro y cinco subcategorías, respectivamente: 1) Reconocer la realidad y sus retos: a) El significado de la muerte en el contexto del nacimiento difiere de otros escenarios; b) Aceptación de diferentes tipos de duelo; c) Conocimiento especializado y disponibilidad emocional; d) Limitaciones institucionales. 2) Identificando hipótesis de soluciones viables: a) Convirtiendo la muerte en un tema transversal; b) Contacto gradual y seguro con la experiencia; c) Espacios para la expresión de sentimientos; d) Grupos de estudio multidisciplinarios; e) Estímulo a la participación activa: el profesional como agente de cambio.

### Reconocer la Realidad y Sus Retos: El Significado de la Muerte en el Contexto del Nacimiento Difiere de Otros Escenarios

Los datos indican que la experiencia de la muerte en el contexto del nacimiento impacta a los profesionales de la salud de manera singular, diferenciándose de otras pérdidas. Esto se debe al significado simbólico del inicio de la vida, junto con la frecuente asociación con las desigualdades sociales y las fallas en la atención, lo que atribuye a la muerte neonatal/materna una carga emocional específica.

*La muerte en el contexto del nacimiento es algo casi desestabilizador [...] cuando ocurre en un centro obstétrico [...] y todo ese espacio está preparado para la vida, y de repente se presenta una situación de muerte, es algo muy delicado y a veces difícil de manejar [...] tanto que la gran mayoría huye [...] nadie quiere tener esa responsabilidad.* (DO2)

*Nos damos cuenta de que la muerte en el contexto del parto está directamente relacionada con los indicadores sociales. Refleja la calidad de la atención sanitaria para esta población, las interseccionalidades, porque sabemos que mueren más mujeres negras, indígenas, de clase baja [...] y, a pesar de que contamos con políticas públicas [...] sabemos que estamos muy lejos de alcanzar los objetivos, especialmente en lo que respecta a la mortalidad materna [...]* [que] *son, en su gran mayoría, muertes evitables.* (DO6)

### Reconocer la Realidad y Sus Retos: Aceptar Diferentes Tipos de Duelo

El contexto del nacimiento implica diferentes tipos de duelo, que varían según el momento de la muerte (inicio del embarazo, final del embarazo, posparto), el tipo de muerte (materna, fetal o neonatal) y sus causas (espontáneas, previstas o esperadas, errores en la identificación precoz de afecciones y manejo, entre otras).

Además, el duelo es experimentado por diferentes sujetos (papás, mamás, hermanos, abuelos, profesionales de la salud, entre otros) y está asociado al significado que el fallecido tenía para cada miembro de la familia.

*Después del curetaje, acabamos sin tratar [a la mujer] [..] A menudo, ella permanecerá hospitalizada un tiempo antes del procedimiento [...] viviendo ese duelo, viviendo esa pérdida y nadie le presta atención. Es real, no hay nadie que vaya allí a darle apoyo y respaldo porque: “bueno, es muy pronto, ni siquiera ha crecido la barriga”.* (DO2)

*Una vez acompañé una muerte materna [...] y vi llegar a la hermanita de diez años y pedir que le dejaran sostener* [al bebé]*, asumiendo ya el papel de madre, una niña de diez años. Ella también perdió a su madre, ¿sabes?* (DO9)

*[...] la muerte de un bebé es cuando pierdes un sueño. [...] Ahora bien, cuando perdemos a la madre, creo que es muy complicado, sobre todo para el padre que se queda y si además queda el bebé. [...] Es como si se borrara una historia. Entonces duele de todas las formas, va a doler la muerte de un anciano, de un bebé, de un niño, de un adolescente, pero creo que son dolores que tienen connotaciones diferentes porque van a tener sueños diferentes, implicaciones diferentes, expectativas diferentes.* (DO11)

### Reconocer la Realidad y Sus Desafíos: Conocimiento Especializado y Disponibilidad Emocional

La disponibilidad emocional que se requiere para afrontar la muerte y el sufrimiento de las familias representa un reto significativo para los profesionales, ya que exige un manejo cuidadoso de los sentimientos, las experiencias y las creencias personales, así como el dominio de competencias técnicas específicas.

*Tratar con alguien que se enfrenta a la muerte es saber silenciar tu propia experiencia de la muerte [...] Me gustaría que alguien me dijera que aún soy joven* [para tener un hijo]*, pero la empatía no se trata de eso, la empatía no se trata de hacer lo que tú querrías que te hicieran a ti, la empatía es hacer lo que esa persona necesita que se haga con ella. Por lo tanto, comprender esta dinámica del contexto de la persona, callar y hacerlo es fundamental.* (DI3)

*Veo que hay recetas básicas o conductas protocolizadas que seguimos [...] Por ejemplo, si tienes una madre que ha perdido un bebé, no la pones en la sala con las otras madres [...]. Cuando un bebé nace* [muerto]*, siempre animas a la madre a que levante al bebé y pueda reconocerlo en la despedida [...] Pero a veces [...] su respuesta es no. Entonces esperas, le das un poco de tiempo [...] Y, a menudo, cambian de opinión [...]. Así que tenemos formas de intentar aliviar ese dolor [...] pero creo que los profesionales deben mirar más allá, para intentar comprender lo que las personas están viviendo.* (DO9)

*Tuvimos una experiencia muy dura con una madre que sabía que el bebé no sobreviviría muchas horas después del parto, y ella tenía otros hijos. Entonces, los otros niños, junto con el papá y la mamá, escribieron una cartita [...] y, cuando ella tuvo al bebé, este vino a nosotros, con vida, para que lo dejáramos ir. [...] Le pusimos toda la ropita [...] y le leímos la carta* [al bebé] *[...]* [y] *se lo quedó en brazos [...]. No es fácil,* [porque] *estás despidiéndote de un niño que ni siquiera es tuyo.* (DO11)

### Reconociendo la Realidad y Sus Retos: Limitaciones Institucionales

Para los participantes en la investigación, la alta rotación de profesionales en las instituciones sanitarias, motivada por los bajos salarios, conlleva una sobrecarga de trabajo, la mecanización de la asistencia y el debilitamiento de la cohesión entre los equipos. En consecuencia, estos factores dificultan el aporte humanizado del duelo, además de aumentar la frecuencia de muertes en el entorno laboral.

*La semana pasada estábamos hablando de que la mortalidad materna está aumentando en las estadísticas y [...]* [eso] *no podía suceder, porque la mortalidad materna es un error, es una falla [...] ¿Y qué vemos? Hay muchos cambios de equipo [...] porque el salario no compensa [...]* [el valor es] *un tercio, en comparación con trabajar en el estado, nadie se queda.* (DO2)

*Yo trabajo mucho en la atención primaria y* [allí] *esto es algo aún más* [lejano]*, porque parece que ya hay que pasar a otro tema: “vamos a derivarlo al psicólogo, esto ya no es de mi competencia”, ¿sabes? Es una evasión [...] Entonces, en el hospital, ya tenemos poca preparación [...], [pero] creo que en la atención primaria,* [es] *aún menos.* (DO6)

*Escuchamos mucho: “ni siquiera tengo tiempo para cuidar a los vivos, ¿voy a cuidar a los que murieron?”. No es que los que murieron no sean importantes, pero no tengo tiempo. Y ahí creo que se forma un círculo vicioso [...] entonces, cuando tengo tiempo, ya ni voy porque ni siquiera lo hago en el transcurso de las cosas que tengo que hacer.* (DO12)

### Identificando Hipótesis de Soluciones Viables: Convertir la Muerte en un Tema Transversal

Teniendo en cuenta el alcance del tema y el impacto que tiene en la asistencia, los participantes concluyeron que el tema de la muerte debería tratarse de manera transversal en la formación en salud.

*Creo que la formación siempre debe tener, de manera transversal,* [y en] *todas las disciplinas, algunos contenidos relacionados con la muerte y el cuidado de las personas y la familia, porque es algo que tiene que ser constante.*

*[...] Tener una propuesta transversal que aborde los distintos tipos de muerte no es un curso de especialización, pero es lo mínimo que el estudiante debe tener en cuanto a conceptos, experiencias y estrategias pedagógicas para ir madurando.* (DO7)

*En el contexto del nacimiento, cuando se produce un aborto [...] también existe el aborto ilegal. [...]* [Y] *estas personas acuden a nosotros en los servicios y también han pasado por un proceso de duelo, de muerte, de pérdida, de juicio social [...] de salud psicosocial. Entonces, si pienso en trabajar esto en la formación, creo que es un tema bioético* [transversal] *que forma parte de ella.* (DO8)

### Identificación de Hipótesis de Soluciones Viables: Contacto Gradual y Seguro con la Experiencia

En cuanto al aprendizaje, se destacó la importancia de proporcionar un contacto gradual y realista con el tema de la muerte, para que los estudiantes comprendan su complejidad y desarrollen las habilidades necesarias para afrontarla.

*Vi niños nacer sin cerebro [...], vi madres que sabían que el niño ya había fallecido y estaban esperando el momento del parto para dar a luz [...] y eso lo viví en la licenciatura. ¿Cuál fue mi beneficio? Había dos enfermeras obstétricas que me acompañaban en la época de la pasantía [...]* [y] *ellas me dieron todo el apoyo, además del emocional, el técnico, yo las acompañaba, hacía todo el trabajo con ellas. Entonces eso fue muy importante y también me aportó madurez profesional.* (DO7)

*Todo el mundo recuerda la primera muerte que acompañó [...] y eso es lo que nos da seguridad, nos aporta conocimiento y reflexión. [...] Quien ha perdido a alguien recuerda que las palabras realmente quedan en su memoria [...], lo que fue bueno [...] y las palabras que [nos] hicieron derrumbar. Por eso hay que tener mucho cuidado al hablar.* (DO9)

### Identificación de Hipótesis de Soluciones Viables: Espacios para dar Rienda Suelta a los Sentimientos

Se destacó la importancia de los grupos de apoyo, en los que profesores y alumnos pueden expresar sus sentimientos relacionados con el duelo, compartir experiencias y participar en actividades conjuntas de educación continua, con el fin de desarrollar competencias técnicas y sensibles para el manejo adecuado de situaciones que involucran la muerte y el duelo.

*A institución, teniendo en cuenta los recursos, le resulta muy difícil contar con un profesional vinculado a ti. El psicólogo no podrá darte apoyo porque va en contra de sus propios principios tener vínculos profesionales allí. Buscar un profesional externo, la mayoría no tiene dinero para pagarlo. Y la institución tampoco. Entonces, ¿qué es lo que siempre he visto? Creas tus pequeños grupos de discusión sobre los problemas vividos [...] para aportar tanto teoría como intercambio de experiencias para nuevos enfrentamientos. Pero la gran mayoría de los grupos siempre son así: “no tengo tiempo, no tengo tiempo”.* (DO7)

*Me sorprendió mucho descubrir a través de mi tesis [...] que los profesionales en general tienen muchas dificultades para lidiar con la muerte, sobre todo para dar la noticia [...] a los familiares. [...] entonces me di cuenta [...] de que los profesionales sufren mucho [...]. Y cuando estas muertes son constantes en tu trabajo, el sufrimiento es aún mayor. [...] Por lo tanto, creo que es necesario ofrecer este acompañamiento a los profesionales, que tampoco tienen momentos para la reflexión.* (DO10)

### Identificación de Hipótesis de Soluciones Viables: Grupos de Estudio Multidisciplinarios

También se sugirió la realización de momentos de intercambio multidisciplinario en las universidades, con el objetivo de promover la comprensión de las diferentes perspectivas sobre la muerte y cómo afrontarla, favoreciendo la superación de las posibles dificultades inherentes a cada área de actuación.

*Creo que el apoyo de los propios colegas, al menos en nuestro departamento [...] tienes un grupo de profesores, que se reúne constantemente [...] todos tienen doctorado, posdoctorado, muchos cursos.... Veo un intercambio muy saludable de experiencias, de planes, de cómo abordar al estudiante. Puede que no te dé un apoyo del 100 %, pero entiendo, desde mi punto de vista, que es saludable y ayuda mucho.* (DO7)

*Esta ronda de conversación de hoy aquí, qué buena cosa incluso para nosotros que estamos participando [...]. Esto debería ocurrir más a menudo, necesitamos hablar más de este tema en la sociedad en general, también en la formación y la asistencia.* (DO10)

### Identificación de Hipótesis de Soluciones Viables: Estímulo a la Participación Activa: El Profesional Como Agente de Cambio

Dos participantes destacaron la importancia de promover la “agencia” del profesional de la salud en el proceso formativo, animándolo a adoptar una postura proactiva ante los conflictos entre la formación recibida, las necesidades de la población atendida y las normativas institucionales vigentes.

*Algo que yo añadiría sería comprender las limitaciones institucionales [...] “No puede entrar”, “no puede ver”, “no puede llevarse” [...] está bien que toda la familia no pueda ver a un niño que está en la UTI [...] pero, ¿está bien que tengamos que enviar a un niño a otro lugar [...] y sacarlo de esa pequeña cuna? [...] Creo que entender las limitaciones institucionales en estos términos [...] es algo importante [...]* [para] *percibirse como un transformador potencial de estas reglas, ¿sabes?* (DI3)

*Nos forman para trabajar de una manera legal y vienen las reglas de la institución [...]. Te paralizas, porque eres una buena persona. Has aprendido a seguir las reglas, no solo porque son necesarias, sino porque también hablan de nuestro contrato social de mantener una práctica que sea coherente, ¿no? [...] La agencia significa precisamente que tú creas las reglas desde una perspectiva [...] de dialogar con esas reglas y crear una nueva forma de vida, una nueva forma de cuidar.* (DO5)

## DISCUSIÓN

Se observa que los docentes y los estudiantes viven situaciones de muerte en el contexto del nacimiento a lo largo de su formación. Sin embargo, debido a la asociación de este escenario con el comienzo de la vida, el significado de la muerte adquiere connotaciones específicas que influyen en la forma de afrontarla tanto por parte de los profesionales como de los dolientes. Por ello, el acompañamiento del duelo en este contexto presenta complejidades, ya que implica factores simbólicos, creencias e injusticias sociales que a menudo se descuidan en los procesos formativos, lo que da lugar a una preparación insuficiente y una disponibilidad emocional limitada para conducir adecuadamente el proceso de pérdida de acuerdo con las necesidades singulares de cada persona en duelo (testimonios de la primera y segunda subcategoría).

Los estudios destacan que la pérdida perinatal a menudo no es reconocida por la sociedad, ya que los padres no tienen la oportunidad de convivir con su hijo durante mucho tiempo. Este hecho fomenta en el imaginario colectivo la creencia de que el sufrimiento es menor en estos casos y que los padres no necesitan cuidados específicos^(8,9,11)^. En consecuencia, los padres en proceso de duelo se ven impedidos de mostrar su dolor en público, al tiempo que su maternidad/paternidad es invalidada por el entorno, incluidos los profesionales de la salud.

Una acogida eficaz requiere prácticas basadas en el diálogo y la construcción de vínculos. Un manual de educación para la muerte sugiere que los profesionales de la salud deben tener en cuenta seis elementos para comprender el proceso de duelo de una persona: 1) quién era el fallecido en la vida del doliente y qué representaba; 2) la naturaleza del vínculo emocional entre ellos; 3) las circunstancias de la muerte y la forma en que el doliente recibió la noticia; 4) antecedentes de duelos complicados anteriores al vivido; 5) la personalidad del doliente; 6) y los factores sociales involucrados, como las influencias culturales, étnicas, religiosas y la presencia de una red de apoyo^(16)^. Por lo tanto, deja explícita la importancia de que los profesionales desarrollen habilidades de “silencio y empatía” para escuchar el dolor del otro, a fin de ser capaces de “mirar más allá de las circunstancias” y prestar una asistencia que vaya más allá de los protocolos institucionales preestablecidos (comentarios de la tercera subcategoría).

La realización de prácticas de cuidado complejas, como el acompañamiento en el duelo, requiere tiempo, capacitación y la articulación de los equipos de salud. En consecuencia, estas prácticas se ven directamente afectadas cuando hay sobrecarga de trabajo, rotación profesional y mecanización de la asistencia (factores mencionados en la cuarta subcategoría). Un estudio descriptivo realizado en 35 unidades de salud familiar en un estado de tamaño medio de Brasil identificó que la alta rotación de los profesionales y la falta de capacitación especializada repercuten en los servicios prestados por los equipos, lo que da lugar a evaluaciones insatisfactorias por parte de los usuarios en casi todos los ítems incluidos en la *Primary Care Assessment Tool* (PCATool-Brasil)^(17)^.

Otro estudio^(18)^, realizado con 166 enfermeros, indicó que la rotación en las instituciones de salud está relacionada con cinco factores: el apoyo de los jefes, la recompensa, el confort físico, el control/presión y la cohesión del equipo. Los datos indican que el aumento de la “recompensa” es el factor más eficaz para reducir la intención de rotación, mientras que la disminución del confort físico y la baja cohesión del equipo la aumentan, lo que es coherente con los testimonios de la subcategoría “limitaciones institucionales”. Curiosamente, estos factores también se han asociado en otras investigaciones al desarrollo de trastornos psíquicos en los profesionales, como el agotamiento, la ansiedad y la depresión, principales causas de bajas y abandonos prematuros del trabajo^(17,19,20)^.

Para superar los retos identificados, los participantes en el presente estudio reflexionaron sobre la importancia de que el tema de la muerte se desarrolle de manera transversal en la formación en salud, permitiendo a los estudiantes identificar y desarrollar gradualmente las habilidades necesarias para afrontarla de manera asistida. También destacaron la importancia de que existan espacios para el diálogo, la aceptación del duelo y la expresión de los sentimientos, tanto en el ámbito académico como en el laboral, además de la creación de grupos de estudio multidisciplinarios que estimulen una postura activa en el proceso de atención en salud.

Estas reflexiones encuentran eco en un estudio cualitativo de 2025 con estudiantes y miembros del cuerpo universitario de una universidad de Bruselas (Bélgica), que participaron en la *Compassionate Week*, un festival sobre la muerte, el duelo y las enfermedades graves. Los participantes relataron que acudieron al festival como una forma de lidiar con pérdidas personales, apoyar a personas cercanas y ampliar sus conocimientos sobre el tema. El evento se percibió como un espacio seguro para la expresión emocional y el intercambio de narrativas, lo que contribuyó a ampliar la comprensión de la muerte y el duelo como dimensiones inherentes a la vida, incluso en el contexto académico, además de favorecer la normalización del diálogo sobre la finitud y el fortalecimiento del apoyo emocional^(21)^.

Estas necesidades se alinean con un estudio que analizó la presencia de disciplinas relacionadas con la educación sobre la muerte, la pérdida y el duelo en 103 cursos de medicina y enfermería en Brasil, que identificó que el 83 % de los cursos abordan la muerte en disciplinas obligatorias, pero solo el 28 % contemplan contenidos relacionados con la educación sobre la muerte, la pérdida y el duelo, sin tratarla nunca como tema central. Estos datos evidencian la fragilidad y la urgencia de una reformulación de los procesos formativos de las profesiones de la salud, para que situaciones complejas e inherentes al ejercicio profesional, como la muerte, se profundicen en los estudios de grado, ofreciendo a los estudiantes la oportunidad de comprender los matices técnicos, simbólicos y emocionales que implican^(7)^.

Además, la relevancia de un aprendizaje detallado sobre cómo afrontar la muerte quedó patente en un estudio realizado en Inglaterra, que investigó la experiencia de padres cuyos bebés habían fallecido antes durante el después del parto. Los resultados demostraron que la terminología utilizada por los profesionales para referirse a la muerte del bebé tiene un impacto significativo en la salud de los padres: aquellos a quienes se les informó que estaban “perdiendo a su bebé”, en lugar de “sufriendo un aborto espontáneo”, mostraron una mayor preparación para afrontar el trabajo de parto, el nacimiento del bebé muerto y para verlo y sostenerlo. Según los autores, esta terminología valida la pérdida, facilitando la despedida y la elaboración del duelo^(22)^. Estos hallazgos corroboran las declaraciones de la subcategoría “contacto gradual y seguro con la experiencia”, que destacan la importancia de la acogida y las palabras utilizadas por los profesionales en el momento de transmitir la noticia de una muerte.

Los autores defienden que el contacto con la terminalidad de la vida provoca una ruptura en las defensas del ego de los estudiantes, docentes y profesionales de la salud, obligándolos a afrontar las emociones relacionadas con la pérdida y el desapego^(10,23)^. Esto propicia la reflexión sobre la inevitabilidad de la muerte y la asunción de las dificultades para afrontarla, incluyendo creencias, conceptos y prejuicios. El acercamiento continuo y asistido al tema naturaliza la muerte en el ámbito académico y profesional, facilitando el intercambio de conocimientos para una mejor preparación^(7,9,22)^. El testimonio de DO7, en la misma subcategoría, reitera esta perspectiva al afirmar que el enfrentamiento supervisado de la muerte, durante la formación académica, le permitió desarrollar su confianza y madurez para lidiar con estos procesos.

En la concepción freireana, la escucha y el diálogo constituyen la base de las relaciones humanas. No hay encuentro sin diálogo, así como no hay respeto sin escucha. A través de la dialogicidad surgen contradicciones y límites de las situaciones vividas, revelando factores ocultos que sustentan las interpretaciones de la realidad. La reflexión conjunta permite a los individuos conocer y trascender los límites de su propia singularidad, encontrando nuevas formas de ser y estar en el mundo(24,25,26).

Esta lógica se aplica a los espacios destinados a la expresión del duelo (mencionados en la tercera subcategoría), que ayudan a los estudiantes a afrontar los retos emocionales de la formación, aportando beneficios como la reducción del estrés, el aumento de la productividad y la concentración, la mejora de la empatía y una mayor capacidad para lidiar con situaciones estresantes, como la muerte^(26)^. Un ejemplo de estos espacios son los *Death Cafés*, una iniciativa originaria del Reino Unido y actualmente presente en más de 90 países, que son encuentros comunitarios informales destinados a compartir experiencias de pérdida y reflexiones sobre la finitud. En estas actividades, los participantes pueden ampliar sus conocimientos emocionales y prácticos sobre la muerte y el duelo, además de fortalecer las redes comunitarias de apoyo, contribuyendo a la promoción de respuestas sociales más integradas al cuidado de las personas en duelo^(27)^. En Brasil, sin embargo, la oferta de este tipo de servicio en las universidades públicas sigue centrada en la región sudeste y está destinada exclusivamente a estudiantes de medicina^(26)^.

En este contexto, la creación de grupos de estudio multidisciplinarios (cuarta subcategoría) es una idea prometedora, ya que estimula el intercambio de experiencias entre personas con diferentes conocimientos y habilidades para superar adversidades similares, lo que permite el crecimiento colectivo y el desarrollo de soluciones acordes con la realidad de cada contexto. Por lo tanto, al abrirse al aprendizaje, los profesionales no solo se sienten más preparados para afrontar situaciones adversas, sino que también aprenden a identificar factores de riesgo para mejorar la asistencia, la calidad de la enseñanza y la eficacia de las políticas públicas^(7,8,9,17,22)^.

Al asumir el protagonismo en su proceso de aprendizaje, los profesionales de la salud desarrollan la conciencia sobre su capacidad de sentir, conectarse y reflexionar, individual y colectivamente, sobre sus derechos y responsabilidades. Incluso ante circunstancias difíciles o aparentemente imposibles, el espíritu investigador, la esperanza y el compromiso con la mejora de la realidad perduran, ya que la adversidad pasa a representar no el fin, sino la frontera entre lo que es y lo que puede llegar a ser (última subcategoría). Así, al dialogar constructivamente sobre los problemas de la vida, los individuos trascienden las limitaciones y hacen que lo “inédito viable” sea una realidad posible^(1,24,25)^.

De esta manera, un proceso formativo centrado en el diálogo amoroso y la relación respetuosa entre educadores y educandos crea un ambiente acogedor, capaz de promover el crecimiento conjunto y capacitar a los involucrados para comprender críticamente su realidad y transformarla activamente. En este sentido, el ejercicio de la búsqueda del conocimiento desde la licenciatura facilita la concienciación de los futuros profesionales sobre la importancia de la educación permanente y el compromiso con la evaluación continua de la práctica a medio del ciclo acción-reflexión-acción^(24,25)^.

### Avances para el Área de la Salud y Limitaciones del Estudio

El estudio contribuye al área de la salud al destacar la complejidad de afrontar la muerte en el contexto del nacimiento, poniendo de manifiesto la necesidad de una formación integral que aborde aspectos técnicos, emocionales y simbólicos. Se destaca la importancia de los espacios de acogida, diálogo y grupos multidisciplinarios para el intercambio de conocimientos, fortaleciendo la preparación de los profesionales y subsidiando políticas que promuevan la salud mental y el bienestar.

Como limitaciones, destacan el contexto pandémico, que dificultó una mayor adhesión de los participantes, y el recorte restringido de la realidad investigada. Por lo tanto, se recomienda realizar futuras investigaciones sobre el tema en otros contextos formativos, en diferentes períodos y con distintos diseños metodológicos, a fin de permitir la comparación. Se refuerza, además, la importancia de la participación de hombres del ámbito de la salud en investigaciones que impliquen temas delicados y relacionados con la salud de la mujer, considerando que, en esta investigación, solo las mujeres se sintieron impulsadas a participar.

## CONCLUSIÓN

El objetivo de este estudio fue identificar soluciones para preparar a los profesionales de la salud para afrontar la muerte en el contexto del nacimiento, basándose en las experiencias de profesores y alumnos. Los resultados apuntan a la necesidad de que la formación en las profesiones sanitarias tenga en cuenta las especificidades simbólicas y emocionales que rodean a la muerte en este escenario, promoviendo la inclusión transversal de la temática en los planes de estudio. Se defiende un contacto gradual y asistido con el tema, articulado con la creación de espacios de escucha y la formación de grupos de estudio multidisciplinarios, con el fin de favorecer la acogida de los diversos tipos de duelo y fortalecer el protagonismo profesional. El diálogo entre diferentes conocimientos resultó fundamental para reconocer las fragilidades y proponer estrategias compatibles con cada realidad formativa. En este proceso, el referente freireano se mostró un aliado importante, al sostener la necesidad de la escucha, el diálogo y la acción-reflexión-acción como fundamentos para la construcción de prácticas más sensibles, críticas y transformadoras en el abordaje de la muerte en la formación en profesiones de salud.

## DISPONIBILIDAD DE DATOS

Todo el conjunto de datos que apoya los resultados de este estudio fue publicado en el propio artículo.
